# Effects of repeated nalfurafine and VZTK35 treatments on fentanyl/cocaine mixture self-administration in a rat drug-vs.-food choice procedure

**DOI:** 10.1007/s00213-026-07047-2

**Published:** 2026-03-28

**Authors:** Celsey M. St. Onge, Nicholas Heslep, E. Andrew Townsend, Yan Zhang, Matthew L. Banks

**Affiliations:** 1 Department of Medicinal Chemistry, Virginia Commonwealth University, 800 E. Leigh Street, Richmond, VA 23298, USA; 2 Department of Pharmacology and Toxicology, Virginia Commonwealth University, 410 North 12th Street, Richmond, VA 23298, USA; 3 Division of Therapeutics and Medical Consequences, National Institute on Drug Abuse, 3 White Flint North, MDNorth Bethesda 20852, USA; 4 Center for Drug Discovery, Virginia Commonwealth University, 800 E. Leigh Street, Richmond, VA 23298, USA; 5 Institute for Drug and Alcohol Studies, 203 East Cary Street, Richmond, VA 23298, USA; 6 Present address: Department of Translational Medicine, The Scripps Research Institute, 10550 N. Torrey Pines Road, SR-305, La Jolla, CA 92037, USA

**Keywords:** Polysubstance use, Fentanyl, Cocaine, Drug self-administration, Kappa opioid receptor, Rat

## Abstract

**Rationale:**

Polysubstance use, defined as the consumption of more than one addictive substance either simultaneously or sequentially, is an established contributing factor to drug overdose deaths. Almost 80% of opioid-related overdose deaths involve a second substance and 21.6% of these deaths involve cocaine. Beyond increased overdose risk, polysubstance use is also linked to poorer treatment outcomes as well as higher relapse and mortality rates compared to single substance use. Moreover, there are no Food and Drug Administration-approved treatments for polysubstance use disorder.

**Objectives:**

This study determined the effectiveness of two kappa opioid receptor (KOR) agonists, nalfurafine (NLF) and VZTK35, to attenuate fentanyl and cocaine co-use using a fentanyl/cocaine-vs.-food choice procedure in male and female Sprague Dawley rats.

**Methods:**

We characterized fentanyl/cocaine mixture interactions through dose-addition analysis, cost manipulations, and repeated naltrexone treatment and examined the effects of repeated NLF and VZTK35 treatment on fentanyl-, cocaine- and fentanyl/cocaine mixture choice.

**Results:**

Fentanyl and cocaine interactions on drug choice were synergistic and sensitive to cost manipulations but not repeated naltrexone treatment. Repeated NLF and VZTK35 treatments failed to attenuate fentanyl-, cocaine- or fentanyl/cocaine choice up to doses that disrupted operant behavior with effects of both compounds antagonized by nor-binaltorphimine.

**Conclusions:**

While candidate medications are needed for fentanyl and cocaine co-use, these results do not support the further evaluation of these KOR agonists for this purpose. However, these methods provide an empirical preclinical framework for studying fentanyl and cocaine interactions on reinforcement endpoints.

## Introduction

Polysubstance use is defined as the consumption of more than one addictive substance either at the same time (simultaneous/concurrent use) or at separate times within a single 24 h period (sequential use) (Midanik et al. 2007; Hakkarainen et al. 2019; Boileau-Falardeau et al. 2022). Polysubstance use is a well-established factor contributing to drug overdose deaths (Mars et al. 2015). Moreover, the urgency of this public health issue is increasingly being recognized as an important factor in the dramatic increase in opioid-related deaths in North America (Cicero et al. 2020; Peppin et al. 2020; Tori et al. 2020; Compton et al. 2021). Polysubstance use is associated with a three-fold higher-mortality rate when compared to use of a single substance (de la Fuente et al. 2014). For example, almost 80% of opioid-related overdose deaths also involved a second substance (Jones et al. 2018). Of these polysubstance-related overdose deaths, 48% involved a second opioid, 21.6% involved cocaine, 11.1% involved alcohol, and 5.4% involved a psychostimulant other than cocaine (Jones et al. 2018). Furthermore, non-fatal polysubstance use is associated with poorer treatment outcomes and higher rates of relapse (American Psychiatric Association 2022; Staiger et al. 2013; Williamson et al. 2006). Unfortunately, the Diagnostic and Statistical Manual of Mental Disorders Fifth Edition Text Revision (DSM-5-TR) lacks categorical discussion of polysubstance use and therefore offers no specific diagnostic criteria. In summary, polysubstance use results in severe negative health outcomes and its treatment represents an unmet clinical need.

Despite epidemiological evidence of polysubstance use, individual addictive substances have been the focus of most preclinical research. Furthermore, polydrug use often serves as an exclusionary criterion for clinical studies (Crummy et al. 2020) precluding the evaluation of substance use disorder medication effects. Including multiple substances at both the preclinical and human laboratory levels may enhance translatability of candidate medication effects on clinical populations wherein misuse commonly includes multiple substances (Compton et al. 2021). Accordingly, several preclinical models have been developed in efforts to capture the multitude of potential polysubstance use patterns in the clinical population as reviewed extensively elsewhere (Crummy et al. 2020; Corley et al. 2024; Gipson et al. 2025; Joffe et al. 2026). Translation may be further facilitated with preclinical procedures that model additional aspects of the clinical condition such as choice between a drug and an alternative reinforcer. Indeed, preclinical drug-vs.-food “choice” self-administration procedures have demonstrated high concordance with human laboratory drug-vs.-money choice procedures (Banks and Negus 2012, 2017; Thomsen et al. 2013a). Although preclinical drug-choice procedures have previously studied substance mixtures such as heroin/cocaine and fentanyl/methamphetamine (Mello et al. 1995; Negus 2005; Martin et al. 2006; Townsend et al. 2021b), fentanyl and cocaine interactions have not been examined. Therefore, the current study sought to develop a preclinical choice model between a fentanyl/cocaine mixture and a non-drug alternative.

When developing candidate medications for polysubstance use, the kappa-opioid receptor (KOR) agonist and mu opioid receptor (MOR) partial agonist, nalfurafine (NLF), has yielded intriguing pharmacological results in both opioid and cocaine research. First, NLF co-administration with morphine suppressed morphine conditioned place preference and hyperlocomotion and these NLF effects were blocked by the KOR antagonist nor-binaltorphimine (nor-BNI) (Tsuji et al. 2001). Furthermore, NLF did not induce place preference, place aversion, or hyperlocomotion when tested alone in mice (Tsuji et al. 2001; Kaski et al. 2019). In addition, NLF co-administration with oxycodone decreased oxycodone self-administration and conditioned place preference in mice and rats as well as oxycodone self-administration in rhesus monkeys (Townsend et al. 2017; Zamarripa et al. 2020a, 2020b; Zhang and Kreek 2020). Second, NLF pretreatment attenuated cocaine place preference and cocaine discrimination in rats (Mori et al. 2002; Hasebe et al. 2004). NLF produced conditioned place aversion at a dose large enough to also disrupt operant responding (Mori et al. 2002). In contrast, NLF pretreatment potentiated cocaine self-administration in mice (Dunn et al. 2020). Lastly, in both intravenous self-administration studies using rats and rhesus monkeys as well as multiple year-long abuse potential studies in humans, NLF showed no evidence of reinforcing effects, physical dependence, withdrawal effects, or tolerance (Kumagai et al. 2012; Ueno et al. 2013; Nakao et al. 2016; St. Onge et al. 2024). Taken together, these data suggest that NLF warrants evaluation as a medication for polysubstance use that includes opioids and cocaine.

Despite positive preclinical evidence of NLF effects in a variety of preclinical models of opioid and cocaine abuse, a previous drug-vs.-food choice self-administration study suggested otherwise. Specifically, acute NLF pretreatment failed to attenuate fentanyl choice and instead non-selectively decreased operant behavior for both fentanyl and food (Townsend 2021a). These undesirable rate-limiting effects of NLF may be partially attributable to its MOR partial agonist activity. Thus, testing KOR agonist/MOR partial agonist pharmacological compounds with a range of KOR and MOR efficacy may provide further insight into the potential for these compounds as substance use disorder medications. Recently, we synthesized a series of NLF analogs including VZTK35, previously published as “Compound 23” (St. Onge et al. 2024). VZTK35 is a KOR agonist with lower MOR efficacy than NLF (Table S1) (St. Onge et al. 2024). Accordingly, the aim of the present study was to determine the effects of repeated 5-day VZTK35 and NLF treatment on fentanyl/cocaine mixture-vs.-food choice in male and female rats. Fentanyl/cocaine mixture interactions were characterized through dose-addition analysis and environmental manipulations (i.e., varying response cost) previously shown to alter fentanyl choice (Townsend et al. 2021b). VZTK35 and NLF repeated 5-day treatment effects were also determined for fentanyl choice and cocaine choice for comparison. Lastly, repeated 5-day naltrexone treatment effects on fentanyl/cocaine mixture choice were determined as an additional comparison with a clinically available opioid use disorder medication.

## Methods

### Subjects

Sprague-Dawley rats (23 male, 16 female) were acquired from Envigo (Frederick, MD, USA) at approximately 17–18 weeks of age with weights of approximately 300 g for males and 250 g for females at the start of the study. Rats were individually housed in a temperature- and humidity-controlled vivarium programmed on a 12-h light/dark cycle (lights on 6:00 AM to 6:00 PM). Food (Teklad Rat Diet, Envigo) and water were provided *ad libitum* in the home cage. Animal research protocols, maintenance, and enrichment were approved by the Virginia Commonwealth University Institutional Animal Care and Use Committee and in accordance with the “Guide for the Care and Use of Laboratory Animals” 8th Ed. (2011).

### Experimental design

**Fig. 1:**
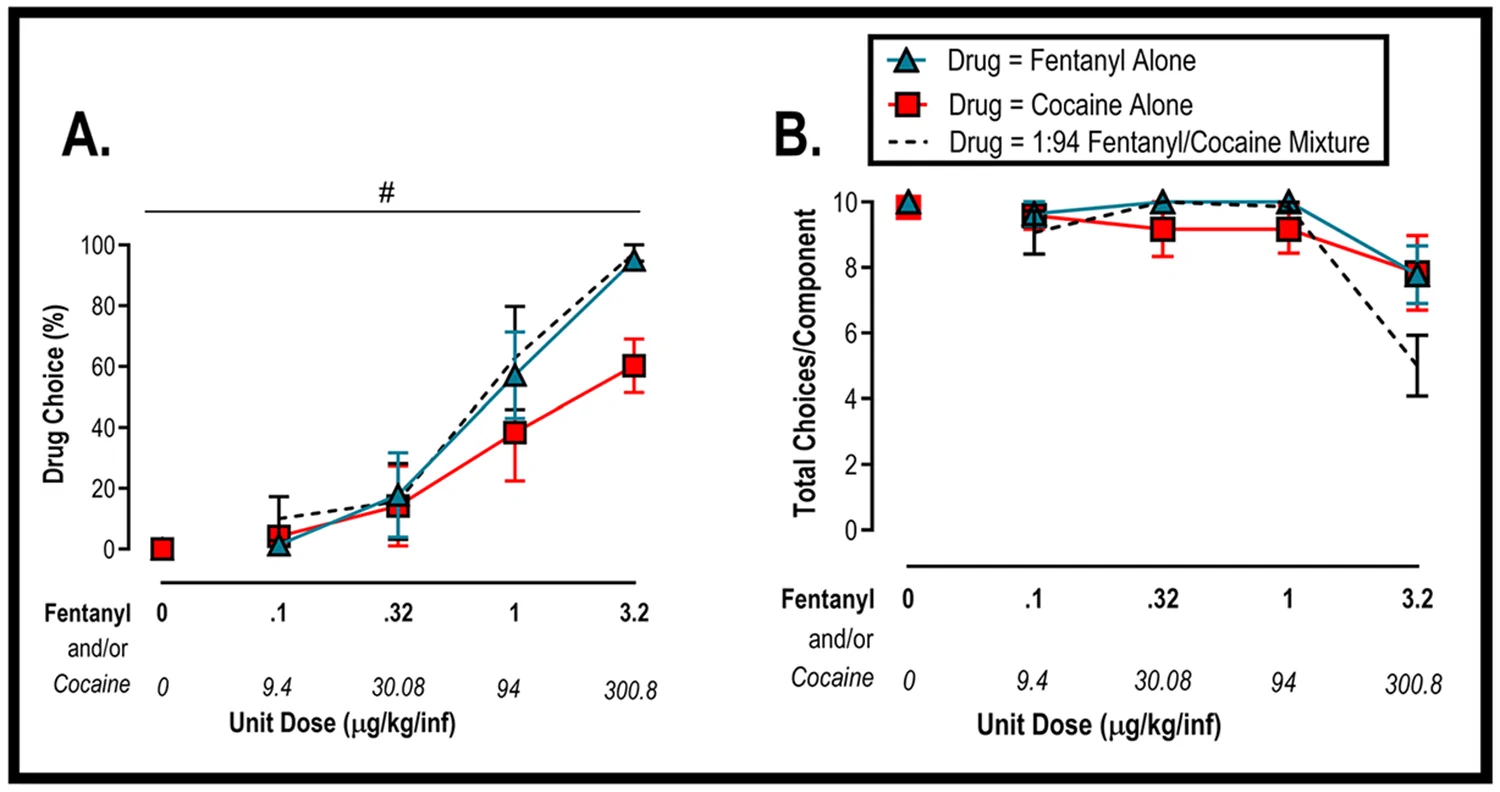
Effect of varying the drug available within session on drug choice in male (*n* = 2) and female (*n* = 4–5) rats; wherein the available doses of fentanyl alone and cocaine alone match those available in the 1:94 fentanyl/cocaine mixture. (**A**) Percent drug choice; drug is 1:94 fentanyl/cocaine mixture (dashed line), fentanyl alone (blue triangles), or cocaine alone (red squares). Abscissa: Intravenous unit doses of fentanyl (bold) and cocaine (italics) in μg/kg/inf. (**B**) Total number of choices completed per component. Abscissa: Intravenous unit doses of fentanyl (bold) and cocaine (italics) in μg/kg/inf. All points represent the mean ± SEM. # (**A**) denotes significant difference between cocaine alone and baseline: drug = 1:94 fentanyl/cocaine mixture regardless of unit dose, defined as *p* < 0.05. See Table S2 in Supplemental Materials for statistics relevant to each panel

**Fig. 2:**
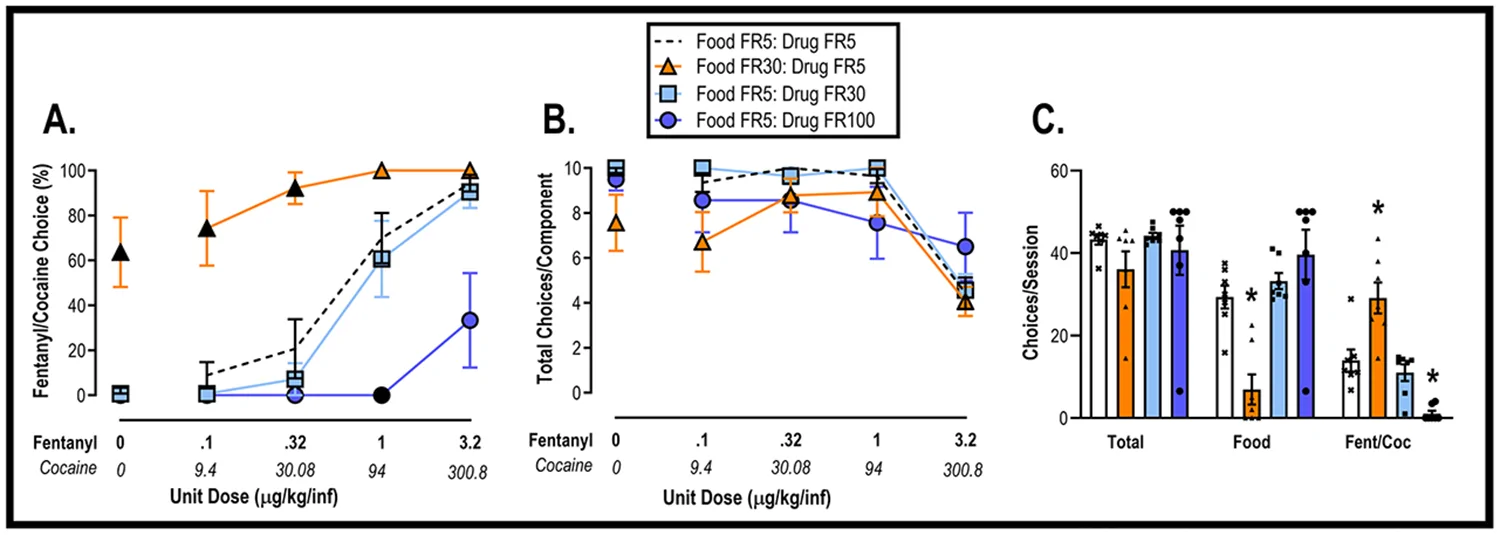
Effect of individually varying the response requirements for food and drug on fentanyl/cocaine mixture choice in male (*n* = 2) and female (*n* = 5) rats. (**A**) Percent 1:94 fentanyl/cocaine mixture. Abscissa: Intravenous unit doses of fentanyl (bold) and cocaine (italics) in μg/kg/inf. (**B**) Total number of choices completed per component. Abscissa: Intravenous unit doses of fentanyl (bold) and cocaine (italics) in μg/kg/inf. (**C**) Total number of choices completed per session. Abscissa: Reinforcer type. All points represent the mean ± SEM. Black filled symbols (**A**) and * (**C**) denote significant difference relative to the baseline: Food FR5: Drug FR5 condition (dashed lines/white bars), defined as *p* < 0.05. See Table S2 in Supplemental Materials for statistics relevant to each panel

**Fig. 3:**
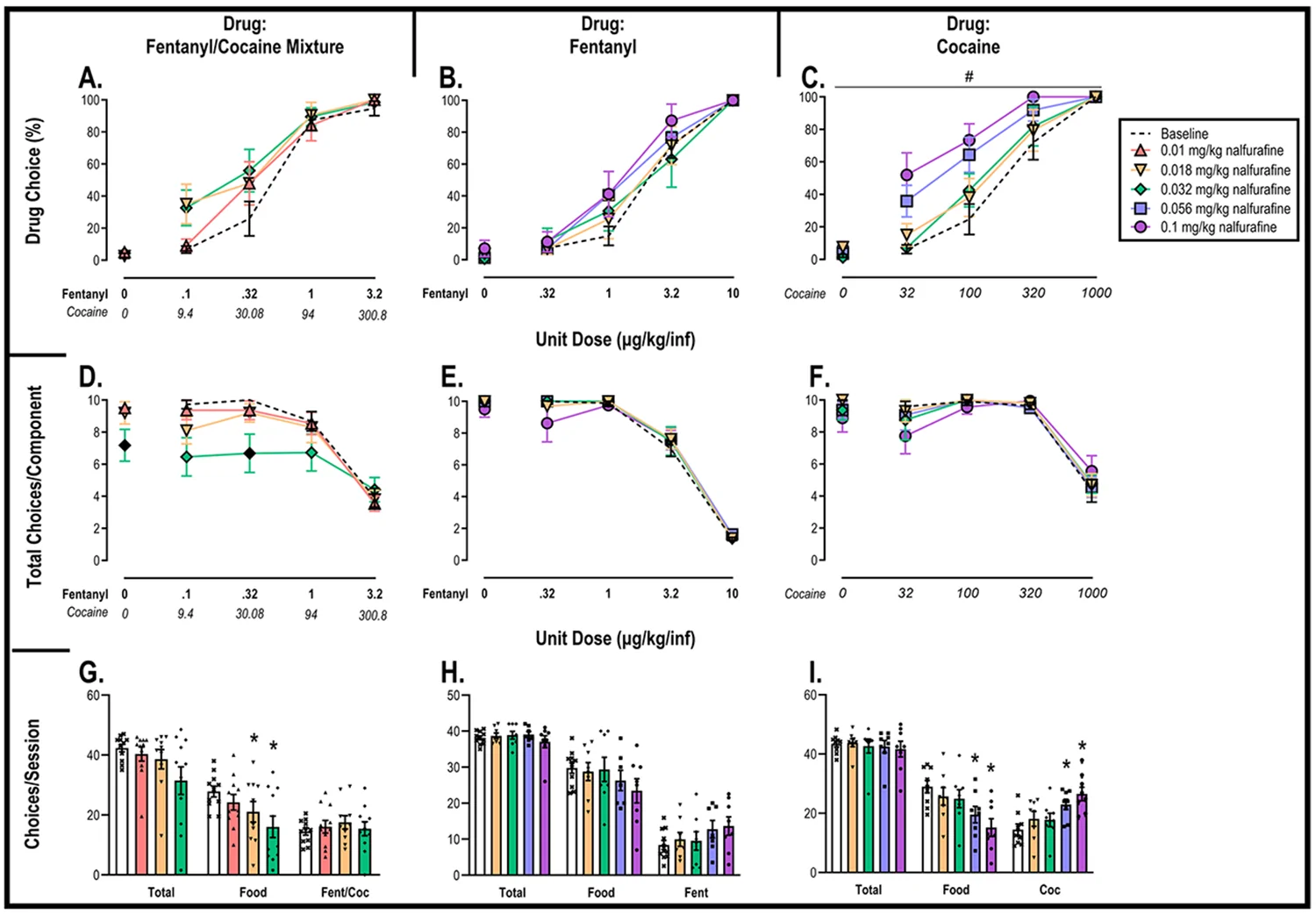
Effects of 5-day repeated subcutaneous injections of nalfurafine on drug-vs.-food choice in male and female rats. Drug was a 1:94 fentanyl/cocaine mixture (**A**, **D**, and **G** [*n* = 5–6 male, *n* = 5 female]), fentanyl (**B**, **E**, and **H** [*n* = 4–7 male, *n* = 4–5 female]), or cocaine (**C**, **F**, and **I** [*n* = 4–5 male, *n* = 4 female]). (**A-C**) Percent drug choice. Abscissa: Intravenous unit doses of fentanyl (bold) and cocaine (italics) in μg/kg/inf. (**D-F**) Total number of choices completed per component. Abscissa: Intravenous unit doses of fentanyl (bold) and cocaine (italics) in μg/kg/inf. (**G-I**) Total number of choices completed per session. Abscissa: Reinforcer type. All points represent the mean ± SEM. Black filled symbols (**D**) and * (**G & I**) denote significant difference relative to baseline (dashed lines/white bars), # (**C**) denotes significant difference between nalfurafine (0.056 and 0.1 mg/kg) and baseline regardless of unit dose, defined as *p* < 0.05. See S2Table in Supplemental Materials for statistics relevant to each panel

**Fig. 4:**
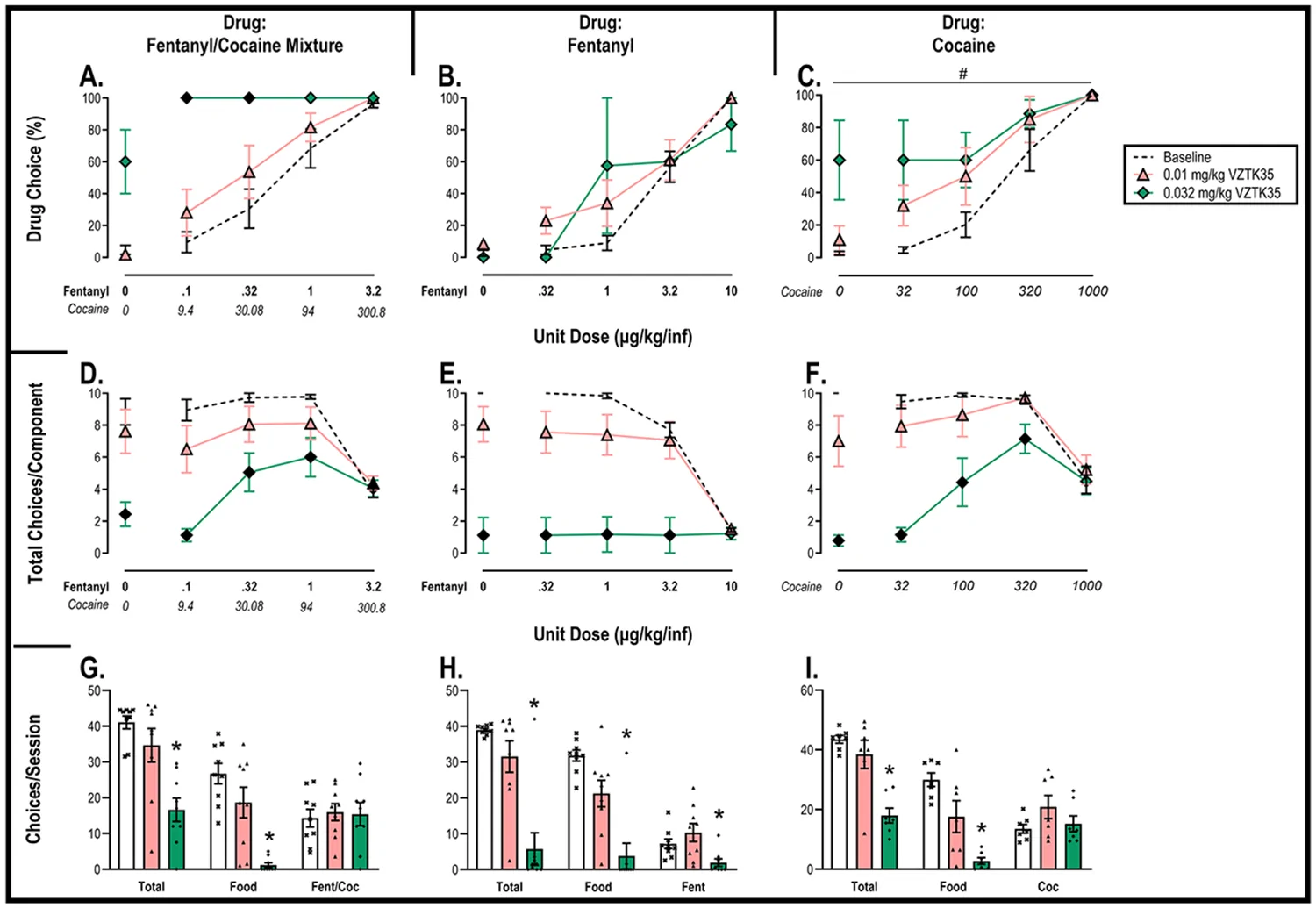
Effects of 5-day repeated subcutaneous injections of VZTK35 on drug-vs.-food choice in male and female rats. Drug was a 1:94 fentanyl/cocaine mixture (**A**, **D**, and **G** [*n* = 3–4 male, *n* = 5 female]), fentanyl (**B**, **E**, and **H** [*n* = 4 male, *n* = 5 female]), or cocaine (**C**, **F**, and **I** [*n* = 3 male, *n* = 4 female]). (**A-C**) Percent drug choice. Abscissa: Intravenous unit doses of fentanyl (bold) and cocaine (italics) in μg/kg/inf. (**D-F**) Total number of choices completed per component. Abscissa: Intravenous unit doses of fentanyl (bold) and cocaine (italics) in μg/kg/inf. (**G-I**) Total number of choices completed per session. Abscissa: Reinforcer type. All points represent the mean ± SEM. Black filled symbols (**A & D-F**) and * (**G-I**) denote significant difference relative to baseline (dashed lines/white bars), # (**C**) denotes significant difference between VZTK35 (0.032 mg/kg) and baseline regardless of unit dose, defined as *p* < 0.05. See S2Table in Supplemental Materials for statistics relevant to each panel

**Fig. 5:**
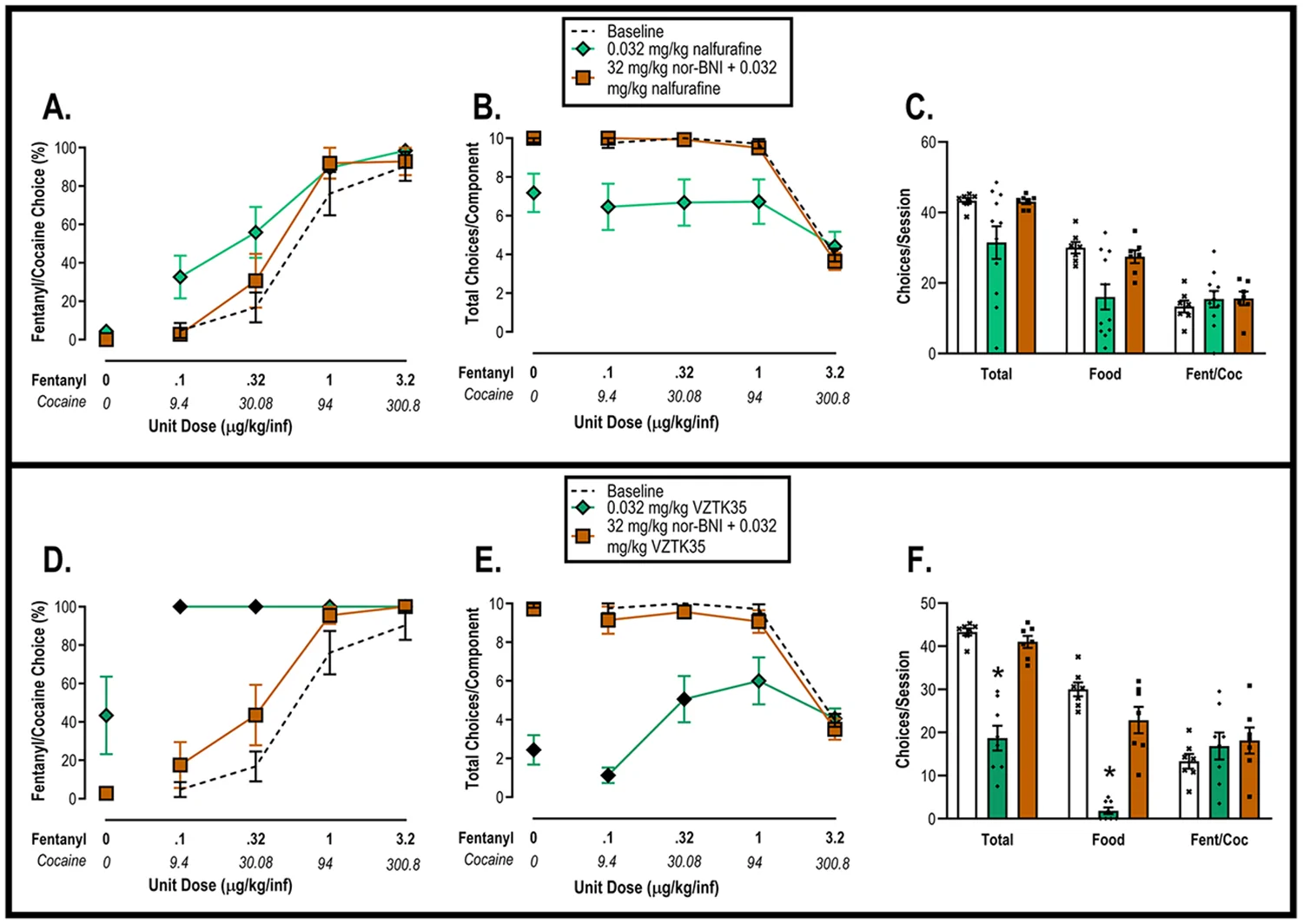
Effect of pretreatment with the KOR antagonist nor-BNI prior to 5-day repeated subcutaneous injections of nalfurafine (**A-C**, top row) and VZTK35 (**D-F**, bottom row) on fentanyl/cocaine mixture-vs.-food choice in male (*n* = 2–6) and female (*n* = 5) rats. (**A&D**) Percent 1:94 fentanyl/cocaine mixture choice. Abscissa: Intravenous unit doses of fentanyl (bold) and cocaine (italics) in μg/kg/inf. (**B&E**) Total number of choices completed per component. Abscissa: Intravenous unit doses of fentanyl (bold) and cocaine (italics) in μg/kg/inf. (**C&F**) Total number of choices completed per session. Abscissa: Reinforcer type. All points represent the mean ± SEM. Black filled symbols (**D & E**) and * (**C & F**) denote significant difference relative to baseline (dashed lines/white bars), defined as *p* < 0.05. See Table S2 in Supplemental Materials for statistics relevant to each panel

**Fig. 6:**
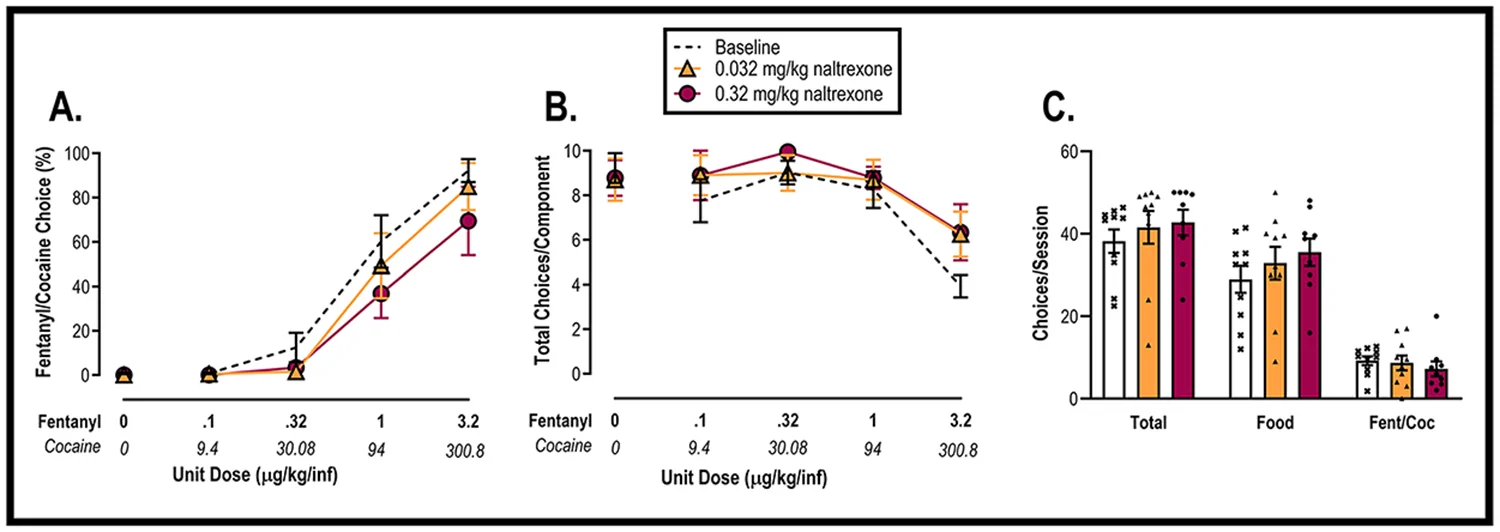
Effect of 5-day repeated subcutaneous injections of naltrexone on fentanyl/cocaine mixture-vs.-food choice in male (*n* = 4–5) and female (*n* = 5) rats. (**A**) Percent 1:94 fentanyl/cocaine mixture choice. Abscissa: Intravenous unit doses of fentanyl (bold) and cocaine (italics) in μg/kg/inf. (**B**) Total number of choices completed per component. Abscissa: Intravenous unit doses of fentanyl (bold) and cocaine (italics) in μg/kg/inf. (**C**) Total number of choices completed per session. Abscissa: Reinforcer type. All points represent the mean ± SEM. See Table S2 in Supplemental Materials for statistics relevant to each panel

Each rat was surgically implanted with a vascular access port (Instech, Plymouth Meeting, PA) and custom-made jugular catheter as previously reported (Townsend et al. 2021b). The 39 rats were divided into three cohorts based on the drug reinforcer that they were trained to self-administer. Cohort 1 (6 male, 6 female) was trained on a 1:94 fentanyl/cocaine mixture and used in Experiments 1 through 5. Cohorts 2 (10 male, 6 female) and 3 (7 male, 4 female) were trained on fentanyl alone and cocaine alone, respectively, and used in Experiment 3. A within-subjects design was used for all experiments and within individual experiments among each cohort. Drug doses were counterbalanced to the extent that adequate prior knowledge of dose ranges and sample sizes allowed. Of note, for cohort 1 (1:94 fentanyl/cocaine mixture), eleven of twelve rats completed training, six of seven rats completed Experiment 1, seven of seven rats completed Experiment 2, eight of eleven rats completed Experiment 3, seven of eleven rats completed Experiment 4, and nine of ten rats completed Experiment 5. In cohort 1, baseline drug choice was re-determined for each experiment to minimize potential carryover effects. For cohort 2 (fentanyl alone), nine of sixteen rats completed Experiment 3, while for cohort 3 (cocaine alone) eight of eleven rats completed Experiment 3. Attrition was a result of poor catheter patency and health issues. Final sample sizes are reported for each experiment (Figs. 1, 2, 3, 4, 5 and 6). Both males and females were included in all experiments to address potential sex differences (Becker and Koob 2016; Jones 2017; Townsend et al. 2019b). Two-hour behavioral testing sessions were conducted five days per week (Mon-Fri) from approximately 1:30–3:30 PM (cohort 1) and 7:30 – 9:30 AM or 10:30 AM – 12:30 PM (cohorts 2 and 3). All experiments occurred during the light phase (Lynch and Roberts 2004; Bass et al. 2010).

### Behavioral apparatus and catheter maintenance

Eighteen modular operant chambers contained within sound-attenuating cubicles (Med Associates, St. Albans, VT) were equipped with two retractable levers, a set of three LED lights (red, yellow, green) mounted above each lever and a retractable dipper cup (0.1 mL) positioned between the two levers as reported previously (Townsend et al. 2021b). The food-associated lever (left side lever) resulted in presentation of liquid food (32% v/v diluted vanilla-flavored Ensure^®^ in tap water, Abbott Laboratories, Chicago, IL, USA) and liquid food availability was signaled by the red LED stimulus light above the lever. The drug-associated lever (right side lever) was paired with intravenous (IV) drug infusions signaled by the green LED stimulus light above the lever and delivered via activation of a syringe pump (PHM-100, Med Associates) located inside the sound-attenuating cubicle. After each behavioral session, IV catheters were flushed with 0.1 mL of gentamicin (4 mg/mL) followed by 0.1 mL of heparinized saline (30 U/mL; cohort 1) or 0.1mL of a solution of heparinized saline (250U/mL) and cefazolin (50 mg/mL; cohorts 2 & 3) after each behavioral session. Catheter patency was verified in all rats at least every two weeks and at the conclusion of the study via instantaneous muscle tone loss following IV methohexital (0.5 mg/0.1 mL) administration.

### Drug-vs.-food choice training

Following surgical implantation of vascular access ports, rats were trained on the terminal drug-vs.-food procedure according to previously published methods (Townsend et al. 2021b). Briefly, rats were first trained to respond for IV drug infusions (inf) under a fixed-ratio (FR) 1/20-s time out schedule of reinforcement. The “drug” varied by cohort. Cohort 1 was trained on a 1:94 fentanyl/cocaine mixture which corresponded to a 1:1 ED_50_ ratio based on the potency of each drug alone in a drug-vs.-food choice procedure based on previously published rat self-administration data (Townsend et al. 2021b). Cohorts 2 and 3 were trained on fentanyl alone and cocaine alone, respectively. The initial drug self-administration training doses were 1 μg/kg/inf fentanyl and 94 μg/kg/inf cocaine for cohort 1, 3.2 μg/kg/inf fentanyl for cohort 2, and 320 μg/kg/inf cocaine for cohort 3. Selected doses were the first half-log dose on the descending limb of the dose-effect function following the peak dose for cohorts 2 and 3 (fentanyl alone and cocaine alone, respectively) (Townsend et al. 2021b). This schedule of reinforcement was in effect until approximately 20 infusions were earned in a single session. Then, the FR requirement was increased either directly (cohort 1) or incrementally (cohorts 2 and 3) to FR 5 and in effect until the number of earned infusions was within 20% of the running mean for three consecutive days with no upward or downward trends.

After completion of drug self-administration training, rats were trained to respond for liquid food presentation under an FR 1/20-s time out schedule of reinforcement in daily 2-h behavioral sessions. This schedule of reinforcement was in effect until approximately 100 food presentations were earned for at least one day. Then, the FR requirement was increased to FR 5 and in effect until the number of food presentations earned was within 20% of the running mean for three consecutive days with no upward or downward trends.

Once drug- and food-maintained responding were both established in isolation, rats were trained on the drug-vs.-food choice procedure as previously reported (Townsend et al. 2021b). Each behavioral session consisted of five, 20 min response components wherein the available unit drug dose increased by 0.5 log unit increments between each component (fentanyl/cocaine mixture 0–3.2 μg/kg/inf fentanyl/0–300.8 μg/kg/inf cocaine; fentanyl alone 0–10 μg/kg/inf; cocaine alone 0–1000 μg/kg/inf) and the food reinforcer remained constant. Drug doses between components varied by increasing the infusion duration. During each behavioral session, both the left (food-associated) and right (drug-associated) levers were extended, and their corresponding stimulus lights illuminated to signal the availability of both food and drug. Response-requirement was an FR 5 for both food and drug. During each response component, up to ten total ratio requirements (food and/or drug) could be completed, and responding on one lever reset the FR for the other lever. Completion of a response requirement began a 20-s time out period with both levers retracted and stimulus lights extinguished. Drug choice was considered stable when the smallest drug dose that maintained ≥ 80% drug choice was within a half-log unit of the running mean for three consecutive days with no upward or downward trends.

### Experiment 1: Effects of varying drug available within-session on drug-vs.-food choice

The effects of varying the “drug” available during daily drug vs. food choice sessions were evaluated in cohort 1. At baseline, drug was a 1:94 fentanyl/cocaine mixture and food was liquid ensure. Here, drug as an independent variable was changed to fentanyl alone and cocaine alone. Both individual drugs were available at the same unit doses present in the mixture (i.e., **1**:*94* **fentanyl**/*cocaine* mixture components [**0**:*0*, **0.1**: *9.4*, **0.32**:*30.08*, **1**:*94*, **3.2**:*300.8* (**μg/kg/inf**:*μg/kg/inf*)], **fentanyl** only components [**0**, **0.1**, **0.32**, **1**, **3.2** (**μg/kg/inf**)], *cocaine* only components [*0*, *9.4*, *30.08*, *94*, *300.8* (*μg/kg/inf*)]). Each of the within-session drug changes was counterbalanced and remained in effect for a period of five days (Mon-Fri) with data from the last two days of the week (Thu-Fri) analyzed and presented. Baseline fentanyl/cocaine mixture choice was obtained the week prior to the start of available drug changes. The effects of varying the drug available within-session were compared to those of varying the training drug between cohorts using baseline fentanyl- and cocaine- choice from cohorts 2 and 3, respectively.

### Experiment 2: Effects of response requirement on fentanyl/cocaine-vs.-food choice

The effects of varying the response requirement for both fentanyl/cocaine mixture infusions and liquid food presentations were individually assessed in cohort 1. At baseline, both fentanyl/cocaine mixture infusions and liquid food presentations were available at FR5. In a counterbalanced order, food was increased to FR30 while drug response requirement remained at FR5, and drug was increased to FR30 while food response requirement remained at FR5. Subsequently, drug was increased to FR100 while food response requirement remained at FR5 in all rats. Each FR change remained in effect for a five-day test week (Mon-Fri), and data are analyzed and presented as the final two days (Thu-Fri). Baseline choice is represented as an average of the Food FR5:Drug FR5 response requirement assessed both the week before and the week after FR manipulations.

### Experiment 3: Effects of repeated nalfurafine and VZTK35 treatment on fentanyl/cocaine mixture-, fentanyl alone- and cocaine alone-vs.-food choice

The effects of repeated 5-day nalfurafine (NLF) and VZTK35 treatment on drug-vs.-food choice were assessed in three cohorts wherein the drug reinforcer varied by cohort (cohort 1, 1:94 fentanyl/cocaine mixture; cohort 2, fentanyl; cohort 3, cocaine). In every case, different NLF doses (cohort 1, 0 [vehicle], 0.01, 0.018, 0.032 mg/kg; cohorts 2 and 3, 0 [no treatment], 0.018, 0.032, 0.056, 0.1 mg/kg) were each evaluated for five consecutive days (to model the clinical scenario wherein substance use disorder treatments are chronically administered). Doses ranged from behaviorally inactive to behaviorally active as determined empirically for all three cohorts guided by published studies (Kawai et al. 2008; Townsend 2021a; St. Onge et al. 2024). Doses of 0.0032 mg/kg VZTK35 (on 1:94 fentanyl/cocaine-mixture-vs.-food choice) as well as 0.01 mg/kg NLF (on fentanyl- and cocaine-vs.-food choice) were piloted in subsets of rats and discontinued to minimize behaviorally inactive doses (data not shown). Once a dose range was established, a counterbalanced dosing order was used for the remainder of the experiment. Doses were administered as a SC injection based on rat weight (as determined weekly) 10 min prior to the start of each behavioral session for the five-day test period (Mon-Fri). Pretreatment times were based on published studies which indicated significant NLF behavioral effects at 10 min post SC injection and lasting beyond 2 h post-injection for both NLF and VZTK35 (Lazenka et al. 2018; Faunce and Banks 2020; Townsend 2021a; St. Onge et al. 2024). These experiments were repeated in the same manner using different VZTK35 doses (0 [vehicle – cohort 1, no treatment – cohorts 2 and 3], 0.01, 0.032 mg/kg). Baseline choice for cohort 1 is represented as the vehicle treatment included in NLF and VZTK35 counterbalances while it is represented as the average of choice in the absence of treatment for cohorts 2 and 3. Vehicle treatment did not affect baseline fentanyl/cocaine mixture-vs.-food choice (data not shown). Data collected on the final two days (Thu-Fri) of each dose test week were used for graphical and statistical analyses.

### Experiment 4: nor-BNI antagonism of nalfurafine and VZTK35 on fentanyl/cocaine-vs.-food choice

To evaluate the necessity of the KOR for the effects of NLF and VZTK35 on fentanyl/cocaine mixture-vs.-food choice in cohort 1, the KOR antagonist nor-BNI was administered (32 mg/kg, IP) 3 days prior (Fri) to the start of the first behavioral session of the new test week (Mon) to account for the slow onset and long duration of action (Endoh et al. 1992; Townsend 2021a). Then, 0.032 mg/kg NLF and 0.032 mg/kg VZTK35 were administered in a counterbalanced order for five consecutive days (Mon-Fri) each as a SC injection 10 min before the fentanyl/cocaine mixture-vs.-food choice behavioral session. Data are presented as the last two days of the week (Thu-Fri). Baseline choice is represented as an average of vehicle treatment the week prior to nor-BNI injection and vehicle treatment the week following the completion of the nor-BNI counterbalance (i.e., three weeks post nor-BNI injection).

### Experiment 5: Effects of naltrexone on fentanyl/cocaine-vs.-food choice

Two naltrexone doses (0 [vehicle], 0.032, 0.32 mg/kg) were determined for a period of five consecutive days (Mon-Fri) in a counterbalanced dosing order on fentanyl/cocaine mixture choice in cohort 1. Naltrexone doses were determined based on prior studies (Paronis and Holtzman 1992; Flynn and France 2021; Walker et al. 2021). SC injections were administered 15 min prior to the start of each behavioral session consistent with prior studies (Flynn and France 2021; Maguire and France 2022). Data are presented as the last two days of the test week (Thu-Fri). Baseline choice is represented as an average of the vehicle treatment in the counterbalance prior to naltrexone injections and the washout week of vehicle injections post-naltrexone.

### Data analysis

The two primary dependent measures for each component of the drug choice session were: (1) percent drug choice, defined as “(number of choices completed on the drug-associated lever/total number of choices completed on both levers) x 100”, and (2) total number of choices completed per component. Other dependent measures included the total number of choices completed per 2 h session and the number of choices for each reinforcer type (food and drug) that make up that total. Data were compared using one- or two-way repeated measures ANOVA and the Geisser-Greenhouse correction was applied as appropriate (Prism 10, GraphPad, La Jolla, CA, USA). A significant ANOVA was followed by a Dunnet’s or Šídák’s post hoc test as appropriate. Statistical significance was defined a priori as *p* < 0.05. See Supplemental Table S2 for all statistical tests and factors, Supplemental Table S3 for sex comparisons, and Supplemental Table S4 for statistical details of Supplemental Figures S1-S11 (i.e., baseline fentanyl/cocaine mixture-vs.-food choice, fentanyl/cocaine interactions between cohorts and comparisons of acute vs. 5-day repeated treatment).

### Dose addition analysis

Fentanyl and cocaine interactions were assessed using both graphical and statistical approaches to dose-addition analysis (Wessinger 1986; Woolverton 1987; Tallarida 2000; Stevenson et al. 2003; Negus 2005; Townsend et al. 2021b). The ED_50_ values for drug choice were defined as the drug dose (alone or in a drug mixture) that produced 50% drug choice using linear regression. Graphically, mean ED_50_ values for cocaine choice alone or as part of a mixture were plotted as a function of ED_50_ values for fentanyl choice alone or as part of a mixture. This data presentation format is known as an isobologram and the line in an isobologram that connects the data points for each drug alone shows predicted data points for drug mixtures inferring additivity of drug effects. Points that fall below the line of additivity are suggestive of synergism (or super-additivity) while points that fall above the line are suggestive of antagonism (or sub-additivity). Statistical evaluation of drug interactions was accomplished by comparing the experimentally determined ED_50_ values for the fentanyl/cocaine mixture (Z_mix_) with the predicted additive ED_50_ value (Z_add_) (Tallarida 2000). Z_mix_ was defined as the total drug dose (i.e., fentanyl dose + cocaine dose) in the fentanyl/cocaine mixture associated with 50% drug choice. Z_add_ was calculated using the equation Z_add_ = *f*A + (1 – *f*)B, where A was the ED_50_ for fentanyl alone, B was the ED_50_ for cocaine alone, and *f* was the fractional multiplier related to the ratio of fentanyl and cocaine concentrations in the mixture according to the equation *f* = (Mixture Ratio)/(Mixture Ratio + (B/A) wherein Mixture Ratio = concentration cocaine/concentration fentanyl. ED_50_ as well as Z_mix_ and Z_add_ values were determined to be different if the 95% confidence limits were nonoverlapping (i.e., *p* < 0.05). Z_mix_ significantly less than Z_add_ indicates synergism (or super-additivity), Z_mix_ not significantly different from Z_add_ indicates additivity, and Z_mix_ significantly greater than Z_add_ indicates antagonism (or sub-additivity).

### Drugs

Fentanyl HCl, (−)-cocaine HCl and (−)-naltrexone HCl were provided by the National Institute on Drug Abuse Drug Supply Program (Bethesda, MD) and dissolved in sterile saline. Nor-BNI di-HCl (synthesized by K. Cheng and K. Rice, National Institutes of Health, Bethesda, MD) was dissolved in 20% DMSO. Nalfurafine HCl and VZTK35 (synthesized in-house) were dissolved in sterile saline (St. Onge et al. 2024). All solutions were passed through a 0.22 μm sterile filter (Millex GV, Millipore Sigma, Burlington, MA) before intravenous (IV), subcutaneous (SC), or intraperitoneal (IP) administration. All drug doses were expressed based on the salt forms listed above and delivered based on individual weights as collected weekly.

## Results

### Experiment 1: Effects of varying drug availability within session on drug-vs.-food choice

The effects of varying drug availability within the self-administered solution are shown in Fig. 1. Figure 1A shows that removing fentanyl, but not cocaine, from the mixture resulted in a significant rightward shift in the drug choice dose-effect function (drug availability: F_1.85,10.16_=11.41, *p* = 0.0028). There was only a main effect of unit drug dose on choices per component (F_1.75,10.48_=18.65, *p* = 0.0005; Fig. 1B). There was no effect of sex on either dependent measure (Table S3).

Figure S1 displays baseline fentanyl/cocaine -vs.-food choice. Baseline drug choice was not significantly different across experiments (data not shown). Figure S1A indicates that fentanyl/cocaine mixture choice was dependent on unit dose with behavior primarily allocated towards food when small drug doses were available and almost exclusive drug choice when the largest unit doses were available (unit dose: F_2.06,20.61_=64.89, *p* < 0.0001). Figure S1B shows choices per component were maximal or near maximal except at the largest drug mixture doses (unit dose: F_2.29,22.90_=36.82, *p* < 0.0001). Figure S1C shows the distribution of choices made by reinforcer type (food and drug) at baseline.

Figure S2 displays baseline fentanyl-, cocaine-, and fentanyl/cocaine choice between cohorts in rats that were trained on those drugs, respectively. Both fentanyl and cocaine alone choice dose-effect functions were significantly rightward shifted compared to the fentanyl/cocaine mixture (unit drug dose: F_5,123_=126.3, *p* < 0.0001; drug available: F_2,29_=12.99, *p* < 0.0001; Figure S2A). The number of choices completed per component was higher in rats that self-administered both fentanyl alone and cocaine alone compared to the fentanyl/cocaine mixture (unit drug dose: F_5,123_=85.94, *p* < 0.0001; drug available: F_2,29_=18.42, *p* < 0.0001; Figure S2B). Figure S2C displays an isobologram for fentanyl/cocaine interactions on drug choice in a fentanyl/cocaine mixture and when fentanyl and cocaine dose-effect functions were determined in separate cohorts trained on those drugs, respectively. The fentanyl/cocaine mixture fell below the theoretical line of additivity (Figure S2C) as the ED_50_ choice values for fentanyl and cocaine in rats without fentanyl/cocaine self-administration history were greater than their respective ED_50_s in the fentanyl/cocaine mixture (Table S5). The Z_mix_ value for the fentanyl/cocaine mixture was significantly less than the Z_add_ value (Table S5), indicative of synergism.

### Experiment 2: Effects of response requirement manipulations on fentanyl/cocaine-vs.-food choice

The effects of varying the response requirements for both food and fentanyl/cocaine are shown in Fig. 2. Increasing the food response requirement from FR5 to FR30 significantly increased fentanyl/cocaine choice (FR: F_1.86,11.16_=37.75, *p* < 0.0001; unit dose: F_2.47,14.82_=28.64, *p* < 0.0001; interaction: F_2.12,12.03_=5.48, *p* = 0.0191; Fig. 2A). Increasing the response requirement for fentanyl/cocaine from FR5 to FR100 decreased fentanyl/cocaine choice (Fig. 2A). There was only a significant main effect of unit dose for choices completed per component (F_2.60,15.57_=54.84, *p* < 0.0001; Fig. 2B). Increasing the food FR to 30 significantly decreased session food choices and significantly increased fentanyl/cocaine choices, whereas increasing the fentanyl/cocaine FR to 100 only significantly decreased drug mixture choices (FR: F_1.58,9.46_=1.61, *p* = 0.2471; reinforcer type: F_1.75,10.52_=74.89, *p* < 0.0001; interaction: F_1.69,10.16_=20.62, *p* = 0.0004; Fig. 2C). There was no effect of sex on any of the dependent measures (Table S3).

### Experiment 3: Effects of repeated nalfurafine and VZTK35 treatment on fentanyl/cocaine mixture, fentanyl alone and cocaine alone-vs.-food choice

The effects of 5-day repeated NLF treatment on drug-vs.-food choice are depicted in Fig. 3. NLF treatments did not significantly alter fentanyl/cocaine-vs.-food choice up to the largest NLF dose examined although there was a trend for NLF to increase fentanyl/cocaine choice (Fig. 3A). The largest 0.032 mg/kg NLF dose decreased choices completed per component (unit dose: F_2.38,23.76_=56.99, *p* < 0.0001; NLF dose: F_1.45,14.47_=5.95, *p* = 0.0194; interaction: F_4.17,39.97_=3.96, *p* = 0.0077; Fig. 3D). This decrease was due to decreased food choices (reinforcer type: F_1.58,15.84_=41.34, *p* < 0.0001; NLF dose: F_1.45,14.47_=5.95, *p* = 0.0194; interaction: F_2.70,25.69_=5.35, *p* = 0.0066; Fig. 3G). Figure 3B shows 5-day repeated NLF administration failed to significantly alter fentanyl choice, choices completed per component (Fig. 3E), or choices completed per session (Fig. 3H). Doses of 0.056 and 0.1 mg/kg NLF significantly increased cocaine choice compared to baseline (NLF dose: F_2.84,19.86_=5.83, *p* = 0.0056; Fig. 3C). NLF did not alter choices completed per component (Fig. 3F) or total session choices but did dose-dependently decrease session food choices and increase session cocaine choices (reinforcer type: F_1.19,9.55_=78.41, *p* < 0.0001; NLF dose: F_1.53,12.20_=0.28, *p* = 0.7003; interaction: F_2.78,18.05_=8.29, *p* = 0.0013; Fig. 3I). Acute NLF pretreatment (day 1 of treatment week) had a significant effect on total choices per component when the drug reinforcer was fentanyl/cocaine mixture (Figure S3) but not fentanyl (Figure S4) or cocaine (Figure S5). Acute NLF pretreatment did not significantly alter drug choice regardless of the drug reinforcer (Figure S3-5). Fentanyl/cocaine mixture and cocaine choice were greater in male rats relative to female rats regardless of the NLF dose (Table S3). When the drug reinforcer was fentanyl/cocaine mixture, repeated NLF injection decreased total completed choices (0.032 mg/kg NLF) and food choice (0.018 and 0.032 mg/kg NLF) in male as compared to female rats (Table S3). When the drug reinforcer was cocaine, repeated NLF injection decreased food choice and increased drug choice in male relative to female rats regardless of NLF dose (Table S3). No other sex differences were detected on any of the dependent measures (Table S3).

The effects of 5-day repeated VZTK35 treatment on drug-vs.-food choice are shown in Fig. 4. VZTK35 increased percent fentanyl/cocaine mixture choice in a dose-dependent manner (unit dose: F_3.21,25.66_=38.20, *p* < 0.0001; VZTK35 dose: F_1.97,15.79_=37.57, *p* < 0.0001; interaction: F_2.98,18.61_=4.72, *p* = 0.013; Fig. 4A). In addition, VZTK35 decreased choices per component (unit dose: F_1.81,14.45_=13.91, *p* = 0.0006; VZTK35 dose: F_1.38,11.04_=26.83, *p* = 0.0001; interaction: F_4.09,30.13_=7.15, *p* = 0.0003; Fig. 4D). VZTK35 treatment also decreased session food choices (reinforcer type: F_1.40,11.23_=19.05, *p* = 0.0005; VZTK35 dose: F_1.86,14.92_=33.21, *p* < 0.0001; interaction: F_2.06,16.48_=19.37, *p* < 0.0001; Fig. 4G). Repeated 5-day VZTK35 administration did not significantly alter fentanyl choice (Fig. 4B). However, 0.032 mg/kg VZTK35 significantly decreased choices completed per component (unit dose: F_2.11,16.89_=41.02, *p* < 0.0001; VZTK35 dose: F_1.89,15.08_=22.25, *p* < 0.0001; interaction: F_3.46,27.66_=15.53, *p* < 0.0001; Fig. 4E). In addition, VZTK35 significantly decreased food, fentanyl, and total choices compared to baseline (reinforcer type: F_1.71,13.65_=70.80, *p* < 0.0001; VZTK35 dose: F_1.89,15.08_=25.25, *p* < 0.0001; interaction: F_2.33,18.60_=13.84, *p* = 0.0001; Fig. 4H). Repeated 5-day 0.032 mg/kg VZTK35 treatment significantly increased cocaine choice (VZTK35 dose: F_1.73,10.38_=6.90, *p* = 0.0145; Fig. 4C). 0.032 mg/kg VZTK35 decreased choices completed per component (unit dose: F_1.44,8.63_=12.85, *p* = 0.004; VZTK35 dose: F_1.51,9.05_=32.36, *p* = 0.0001; interaction: F_3.32,19.94_=8.51, *p* = 0.0006; Fig. 4F). In addition, VZTK35 decreased total and food choices per session without significantly altering cocaine choices (reinforcer type: F_1.32,7.91_=27.30, *p* = 0.0005; VZTK35 dose: F_1.51,9.05_=32.36, *p* = 0.0001; interaction: F_1.52,9.09_=9.85, *p* = 0.0074; Fig. 4I). Acute VZTK35 pretreatment significantly affected total choices made per component but not drug choice when the drug reinforcer was the fentanyl/cocaine mixture (Figure S6) or fentanyl (Figure S7). Acute VZTK35 pretreatment significantly increased cocaine choice and decreased choices per component (Figure S8). When the drug reinforcer was the fentanyl/cocaine mixture, the number of total completed choices was greater in females than males regardless of VZTK35 dose with significantly decreased food choice in males relative to females during repeated 0.01 mg/kg VZTK35 injection (Table S3). No other sex differences were detected on any of the dependent measures (Table S3).

### Experiment 4: nor-BNI antagonism of nalfurafine and VZTK35 effects on fentanyl/cocaine-vs.-food choice

The effects of 5-day repeated NLF injections on fentanyl/cocaine-vs.-food choice determined 3-days after intraperitoneal nor-BNI administration are shown in Fig. 5A-C. The non-significant leftward-trend in fentanyl/cocaine choice during 0.032 mg/kg NLF treatment was absent following 32 mg/kg nor-BNI pretreatment (unit dose: F_2.15,21.51_=63.49, *p* < 0.0001; nor-BNI: F_1.28,12.78_=5.01, *p* = 0.0366; Fig. 5A). Because the interaction of unit dose and nor-BNI was not significant, the analysis defaulted to a one-way repeated measures ANOVA with nor-BNI as a factor collapsed on unit dose (F_1.22,7.34_=3.01, *p* = 0.1214). 32 mg/kg nor-BNI blocked 0.032 mg/kg NLF effects on choices completed per component (unit dose: F_1.93,19.25_=41.42, *p* < 0.0001; nor-BNI: F_1.01,10.08_=2.74, *p* = 0.1287; interaction: F_2.44,12.22_=4.99, *p* = 0.0215; Fig. 5B). 32 mg/kg nor-BNI also blocked 0.032 mg/kg NLF effects on session food choices (reinforcer type: F_1.22,12.21_=64.99, *p* < 0.0001; nor-BNI: F_1.01,10.08_=2.74, *p* = 0.1287; interaction: F_2.03,8.12_=4.81, *p* = 0.0416; Fig. 5C). Nor-BNI effects were observed on the first day of the 0.032 mg/kg NLF treatment (Figure S9). A significant difference in completed choices was detected prior to nor-BNI treatment (repeated 0.032 mg/kg NLF injection only) driven by decreased food choice in males relative to females (Table S3). No other sex differences were detected on any of the dependent measures (Table S3).

Nor-BNI effects on repeated 5-day VZTK35 treatment determined 3-days after nor-BNI administration are shown in Fig. 5D-F. 32 mg/kg nor-BNI blocked the leftward shift in fentanyl/cocaine choice during 0.032 mg/kg VZTK35 treatment (unit dose: F_2.73,19.10_=59.16, *p* < 0.0001; nor-BNI: F_1.67,11.65_=53.01, *p* < 0.0001; interaction: F_2.62,13.11_=7.47, *p* = 0.0045; Fig. 5D). Nor-BNI also blocked 0.032 mg/kg VZTK35-induced decreases in choices per component (unit dose: F_2.22,15.51_=26.51, *p* < 0.0001; nor-BNI: F_1.23,8.63_=57.57, *p* < 0.0001; interaction: F_2.53,14.54_=15.40, *p* = 0.0001; Fig. 5E) and session food choices (reinforcer type: F_1.09,7.60_=40.06, *p* = 0.0002; nor-BNI: F_1.23,8.63_=58.00, *p* < 0.0001; interaction: F_1.97,10.84_=21.80, *p* = 0.0002; Fig. 5F). Nor-BNI effects were observed on the first day of the 0.032 mg/kg VZTK35 treatment (Figure S10). There were no sex differences on any of the dependent measures (Table S3).

### Experiment 5: Effects of naltrexone on fentanyl/cocaine-vs.-food choice

Figure 6 shows the effects of repeated 5-day naltrexone treatment on fentanyl/cocaine-vs.-food choice. Up to 0.32 mg/kg naltrexone, there was no significant effect on fentanyl/cocaine choice (Fig. 6A), choices per component (Fig. 6B), or choices per session (Fig. 6C). Naltrexone did significantly increase choices per component after acute pretreatment only (day 1 of testing week; Figure S11). The number of total completed choices was greater in females than males regardless of naltrexone dose (Table S3). No other sex differences were detected on either dependent measure (Table S3).

## Discussion

This study utilized a fentanyl/cocaine-vs.-food choice procedure in rats to determine the effectiveness of two KOR agonists with varying MOR agonist activity to attenuate fentanyl and cocaine co-use. This work built upon previously reported preclinical models of single substance- and polysubstance choice procedures as well as evaluation of NLF for substance use disorders (Mello et al. 1995; Tsuji et al. 2001; Mori et al. 2002; Hasebe et al. 2004; Negus 2005; Martin et al. 2006; Townsend et al. 2017, 2021a, 2021b, Kaski et al. 2019; Dunn et al. 2020; Zamarripa et al. 2020a, 2020b; Zhang and Kreek 2020). There were five main findings. First, we characterized fentanyl and cocaine interactions on drug choice as synergistic. Second, fentanyl/cocaine choice was sensitive to cost manipulations consistent with preclinical drug-vs.-food and human laboratory drug-vs.-money studies (Nader and Woolverton 1992; Woolverton and English 1997; Hart et al. 2000; Heishman et al. 2000; Negus 2003; Hursh and Silberberg 2008; Cantin et al. 2010; Thomsen et al. 2013a; Lile et al. 2016; Reiner et al. 2021). Third, repeated NLF and VZTK35 treatments failed to attenuate fentanyl-, cocaine- or fentanyl/cocaine-vs.-food choice consistent with acute NLF’s effects on a fentanyl-vs.-food choice procedure and similar to other KOR agonist effects in drug-vs.-food or money procedures (Walsh et al. 2001; Negus 2004; Banks 2020; Townsend 2021a). Fourth, NLF and VZTK35 treatment effects on fentanyl/cocaine choice were mediated by KOR as demonstrated by nor-BNI antagonism (summarized extensively by Zhou et al. 2022) (Zamarripa et al. 2020a; Zhang and Kreek 2020; Townsend 2021a; Zhou et al. 2022). Finally, fentanyl/cocaine choice was insensitive to repeated naltrexone treatment consistent with previous cocaine-vs.-food but not fentanyl-vs.-food choice studies (Spealman and Bergman 1992; Hemby et al. 1996; Townsend et al. 2019a). These results do not support the further evaluation of KOR agonists as candidate medications for fentanyl or cocaine self-administration either alone or in this specific combination. However, the experiments and methods do provide an empirical preclinical framework for studying fentanyl and cocaine interactions on reinforcement endpoints.

### Fentanyl and cocaine interactions were synergistic and sensitive to cost manipulations but not naltrexone

Previous studies examining opioid and psychostimulant interactions on drug self-administration endpoints have reported either additive (heroin/cocaine) or synergistic (fentanyl/methamphetamine or morphine/cocaine) interactions (Spealman and Bergman 1992; Rowlett and Woolverton 1997; Negus et al. 1998; Rowlett et al. 2005; Negus 2005; Smith et al. 2006; Trujillo et al. 2011; Townsend et al. 2021b). The present results are consistent with and extend this published literature demonstrating fentanyl and cocaine interactions were synergistic in a rat drug-vs.-food choice procedure. One limitation of the present study was that only one fixed-proportion fentanyl/cocaine mixture was examined. Thus, the degree to which other fentanyl/cocaine mixtures might result in synergistic, additive, or antagonistic interactions on drug choice remains to be empirically determined. Another way that fentanyl and cocaine interactions were assessed in the present study was by removing either fentanyl or cocaine from the self-administered solution. Surprisingly, the dose-effect function for cocaine alone (i.e., when fentanyl was removed from the fentanyl/cocaine mixture), but not fentanyl alone, was seemingly right-shifted compared to the fentanyl/cocaine mixture. Importantly, while this dose-effect function for cocaine-alone aligns with that of rats trained on cocaine-vs.-food choice (e.g., Cohort 3 baseline; Figure S2) consistent with a rightward shift in potency, larger cocaine doses may be necessary to delineate this from a downward shift indicating decreased reinforcer efficacy. These results suggest that the fentanyl proportion of the mixture was contributing more to the reinforcing effects of the mixture compared to cocaine. Additionally, the dose-effect function for cocaine alone aligns with 0.32 mg/kg naltrexone pretreatment producing a non-significant rightward trend in the fentanyl/cocaine mixture dose-effect function. Previous studies demonstrated that 0.1 mg/kg naltrexone shifted the fentanyl discriminative stimulus and antinociception dose-effect function at least 15-fold (Flynn and France 2021; Paronis and Holtzman 1992; Walker et al. 2021). Thus, these data suggest that naltrexone monotherapy would be insufficient to treat fentanyl and cocaine polydrug use, consistent with recent clinical observations (Xu et al. 2022).

For comparison to NLF and VZTK35 treatment effects on fentanyl/cocaine mixture choice, we determined the effects of manipulating the response requirement for both the fentanyl/cocaine mixture and food. Numerous preclinical rat and monkey studies have demonstrated that both fentanyl-vs.-food and cocaine-vs.-food choice were sensitive to response requirement (i.e., cost) manipulations such that increasing the cost for drug shifted the choice dose-effect function rightward and increasing the cost for food shifted the choice dose-effect function leftward (Nader and Woolverton 1992; Woolverton and English 1997; Negus 2003; Cantin et al. 2010; Thomsen et al. 2013a; Reiner et al. 2021). The present results with a fentanyl/cocaine mixture were consistent with these previous studies examining fentanyl and cocaine choice alone. Larger FR values for the fentanyl/cocaine mixture (FR100) were required to shift the choice dose-effect function rightward compared to the FR values for food (FR30) suggesting that although food and fentanyl/cocaine functioned as economic substitutes in this choice context, they were imperfect substitutes. Additionally, previous studies identified significant decreases in fentanyl and cocaine choice at FR values of 30 and 25, respectively (Thomsen et al. 2013b; Reiner et al. 2021). This indicates that the fentanyl/cocaine mixture may have a greater essential value than either fentanyl or cocaine alone, however additional studies using behavioral economic demand procedures are warranted to make this conclusion. Overall, these nonpharmacological manipulations and naltrexone treatment effects provide an empirical foundation for interpreting NLF and VZTK35 treatment effects on fentanyl/cocaine choice.

### Nalfurafine and VZTK35 failed to attenuate fentanyl-, cocaine- or fentanyl/cocaine mixture choice

Substance use disorder treatment goals are to simultaneously decrease drug use and reallocate that behavior towards non-drug alternative reinforcers (Mello and Stevens 1996; Haney and Spealman 2008; Czoty et al. 2016; Townsend et al. 2020). Preclinical drug choice procedures allow for simplified assessment of these treatment goals. In this study, repeated 5-day NLF and VZTK35 pretreatments failed to attenuate fentanyl/cocaine choice. In fact, VZTK35 increased fentanyl/cocaine choice whereas NLF produced non-significant trends towards increased fentanyl/cocaine choice. VZTK35 and NLF pretreatments also significantly increased cocaine choice, but not fentanyl choice. The VZTK35 and NLF results are consistent with a previous nonhuman primate study examining treatment with the KOR agonist U50,488 on cocaine choice as well as the effect of NLF pretreatment on cocaine self-administration in mice (Negus 2004; Dunn et al. 2020). Furthermore, the present repeated 5-day NLF results were also consistent with published acute NLF pretreatment effects on fentanyl choice and extend those results to repeated NLF dosing regimens (Townsend 2021a). Thus, the VZTK35 effects on fentanyl/cocaine choice seem to be driven mostly by VZTK35 effects on cocaine choice rather than fentanyl choice. These results may indicate that NLF and VZTK35 treatment increases the ability of cocaine but not fentanyl to compete with food as a reinforcer, consistent with a previous study on KOR agonist administration during cocaine-vs.-food choice (Negus 2004). Further study is needed to determine whether this shift in relative reinforcing efficacy is due to an underlying change in food or cocaine reinforcing efficacy.

### The effects of nalfurafine and VZTK35 on 1:94 fentanyl/cocaine choice are mediated by the KOR

32 mg/kg nor-BNI pretreatment blocked VZTK35 effects on fentanyl/cocaine choice and blocked VZTK35 and NLF effects on operant responding. The present results are consistent with the extant literature demonstrating this nor-BNI dose was sufficient to block KOR agonist effects on drug self-administration endpoints following both non-contingent and contingent KOR agonist administration (Zamarripa et al. 2020a; Zhang and Kreek 2020; Townsend 2021a). Based on previous opioid and stimulant drug self-administration studies, nor-BNI was not hypothesized to have any effect on fentanyl/cocaine mixture choice in nondependent rats (Negus et al. 1993, 1997; Glick et al. 1995; Negus 2004; Wee et al. 2009). Consistent with this hypothesis, baseline 1:94 fentanyl/cocaine mixture-vs.-food choice was not significantly different before and after nor-BNI injection (data not shown). Overall, these nor-BNI results support the hypothesis that KOR was necessary for both NLF and VZTK35 pretreatment effects on drug choice.

### Implications of the current results on the neurobiology of fentanyl and cocaine reinforcement

The present results suggest two broad general interpretations regarding the distinct roles of KOR and dopamine in fentanyl and cocaine reinforcement. First, the selective increase in cocaine, but not fentanyl choice by both KOR agonists confirms the extant literature supporting a prominent role of KOR in cocaine reinforcement and a lesser role of KOR in opioid reinforcement (Martinez et al. 2019; Estave et al. 2023; Johnson et al. 2025). Moreover, KOR agonist-induced depression of nucleus accumbens dopamine supports a differential role of dopamine in opioid vs. cocaine reinforcement consistent with the literature wherein while both opioids and cocaine increase nucleus accumbens dopamine, cocaine has a more direct mechanism (Badiani et al. 2011; Leitl et al. 2014). Indeed, the increased cocaine choice was greatest following VZTK35 administration relative to NLF, correlating with the greater MOR/KOR relative potency of VZTK35. Future research could examine additional compounds with different KOR and MOR efficacies in other animal models of the substance use disorder continuum to systematically evaluate the relationship between KOR and MOR co-activation on drug reinforcement. However, the present results do not support the further development of KOR agonists as candidate medications for fentanyl or cocaine self-administration either alone or in this specific combination.

Considering the substantial unmet clinical need posed by polysubstance use disorder, the present study underscores the importance of elucidating a mechanism through which fentanyl/cocaine co-use may be modulated pharmacologically. Accordingly, evaluation of additional compounds with alternative and potentially novel pharmacological mechanisms of action represents an important future research direction. Given the inherent complexity of modeling polysubstance use (for a detailed review, see (Crummy et al. 2020; Corley et al. 2024; Gipson et al. 2025; Joffe et al. 2026)), the coordinated application of multiple preclinical models may be necessary to address this challenge. For instance, while the current study assessed fentanyl and cocaine interactions through concurrent self-administration, the present results may not extend to environmental or pharmacological mechanisms of sequential fentanyl and cocaine self-administration. Additional research is needed to characterize these interactions as well as to more fully capture the multitude of polysubstance use scenarios in the clinical population. The experiments and methods described herein provide an empirical preclinical framework for studying fentanyl and cocaine interactions during concurrent self-administration on reinforcement-related endpoints to aid in achieving this goal.

## Supplementary Material

Supplemental Material

**Supplementary Information** The online version contains supplementary material available at https://doi.org/10.1007/s00213-026-07047-2.

## Data Availability

The data are available from the corresponding author upon reasonable request.

## Highlights

- A fentanyl/cocaine-vs.-food choice procedure was established.
- Fentanyl/cocaine interactions were synergistic.
- Fentanyl/cocaine choice was sensitive to cost manipulations.
- Fentanyl/cocaine choice was insensitive to naltrexone treatment.
- KOR agonists did not attenuate fentanyl-, cocaine-, or fentanyl/cocaine-choice.
