# Supplementary material for: Effects of repeated nalfurafine and VZTK35 treatments on fentanyl/cocaine mixture self-administration in a rat drug-vs.-food choice procedure
Source: Psychopharmacology (Berl). Author manuscript; Available in PMC 2026 Sep 14. (PMC13574490; doi:10.1007/s00213-026-07047-2)
Supplement: Supplementary file 1 [file NIHMS2206589-supplement-Supplemental_Material.docx]

**Supplemental Materials:**

**Effects of nalfurafine, VZTK35 and naltrexone treatments on fentanyl/cocaine mixture self-administration in a rat drug-vs.-food choice procedure**

Celsey M. St. Onge^a,1,#^, Nicholas Heslep^b^, E. Andrew Townsend^b,c^, Yan Zhang^a,b,d,e^, and Matthew L. Banks^b^

^a^Department of Medicinal Chemistry, Virginia Commonwealth University, 800 E. Leigh Street, Richmond, Virginia 23298, USA

^b^Department of Pharmacology and Toxicology, Virginia Commonwealth University, 410 North 12^th^ Street, Richmond, Virginia 23298, USA

^c^Division of Therapeutics and Medical Consequences, National Institute on Drug Abuse, 3 White Flint North, North Bethesda, MD 20852, USA

^d^Center for Drug Discovery, Virginia Commonwealth University, 800 E. Leigh Street, Richmond, Virginia 23298, USA

^e^Institute for Drug and Alcohol Studies, 203 East Cary Street, Richmond, Virginia 23298, USA

^1^Present Address: Department of Translational Medicine, The Scripps Research Institute, 10550 N. Torrey Pines Road, SR-305, La Jolla, CA 92037, USA

^#^Correspondance to Celsey M. St. Onge: Department of Translational Medicine, The Scripps Research Institute, 10550 N. Torrey Pines Road, SR-305, La Jolla, CA 92037, USA, E-mail address: cstonge@scripps.edu

# Supplemental Tables

**Supplemental Table S1. Comparison of nalfurafine (NLF) and VZTK35 as previously published** (St. Onge et al., 2024)

| **Compound** | **NLF** | | | | **VZTK35** | | | |
| --- | --- | --- | --- | --- | --- | --- | --- | --- |
| **Structure** |  | | | |  | | | |
| **Binding affinity***  **(*K_i_*, nM)** | **KOR**  0.19 ± 0.01 | **MOR**  0.72 ± 0.09 | | **DOR**  115.9±20.9 | **KOR**  0.22 ± 0.03 | **MOR**  0.78 ± 0.05 | | **DOR**  7.93 ± 1.02 |
| **Selectivity ratio** | **µ/κ**  4 | | **δ/κ**  610 | | **µ/κ**  4 | | **δ/κ**  36 | |
| **Potency****  **(EC_50_, nM)** | **KOR**  0.26 ± 0.03 | **MOR**  0.52 ± 0.12 | | **DOR**  141.4±14.8 | **KOR**  0.14 ± 0.02 | **MOR**  5.31 ± 0.53 | | **DOR**  16.37±3.25 |
| **Efficacy****  **(E_max_, %)** | **KOR**  95.95±4.13 | **MOR**  30.24±3.73 | | **DOR**  65.68±6.42 | **KOR**  106.5±0.7 | **MOR**  14.22±1.77 | | **DOR**  96.13±6.52 |
| **Relative potency** | **µ/κ**  2 | | **δ/κ**  544 | | **µ/κ**  38 | | **δ/κ**  117 | |

*Binding affinity as determined by radioligand binding assays

**Potency and efficacy as determined by [^35^S]-GTPγS assays

## Supplemental Table S2. Statistical details of main text figures (GraphPad Prism Version 10.3 for Windows)

| **Figure number** | **Factor name** | **Values** | ***p*-value** |
| --- | --- | --- | --- |
| Figure 1A: Effect of varying drug available within-session and unit drug dose on drug choice (%)  Mixed-effects model (REML)  Figure 1A: Effect of varying drug available within-session on drug choice (%)  Mixed-effects model (REML) | **Unit Drug Dose**  **Drug Available**  Interaction  **Drug Available** | **F_1.80,10.80_=27.42**  **F_1.83,10.98_=9.74**  F_2.06,11.08_=2.53  **F_1.85,10.16_=11.41** | **<0.0001**  **0.0042**  0.1231  **0.0028** |
| Figure 1B: Effect of varying drug available within-session and unit drug dose on total choices/component  Mixed-effects model (REML) | **Unit Drug Dose**  Drug Available  Interaction | **F_1.75,10.48_=18.65**  F_1.08,6.48_=2.56  F_1.35,7.28_=2.54 | **0.0005**  0.1571  0.1515 |
|  |  |  |  |
| Figure 2A: Effect of varying drug or food FR requirements and unit drug dose on fentanyl/cocaine choice (%)  Mixed-effects model (REML) | **Cost (FR)**  **Unit Fent/Coc Dose**  **Interaction** | **F_1.86,11.16_=37.75**  **F_2.47,14.82_=28.64**  **F_2.12,12.03_=5.48** | **<0.0001**  **<0.0001**  **0.0191** |
| Figure 2B: Effect of varying drug or food FR requirements and unit drug dose on total choices/component  RM two-way ANOVA | Cost (FR)  **Unit Fent/Coc Dose**  Interaction | F_1.58,9.46_=1.61  **F_2.60,15.57_=54.84**  F_3.69,22.14_=2.77 | 0.2471  **<0.0001**  0.0563 |
| Figure 2C: Effect of varying drug or food FR requirements and reinforcer type on choices/session  RM two-way ANOVA | Cost (FR)  **Reinforcer Type**  **Interaction** | F_1.58,9.46_=1.61  **F_1.75,10.52_=74.89**  **F_1.69,10.16_=20.62** | 0.2471  **<0.0001**  **0.0004** |
|  |  |  |  |
| Figure 3A: Effect of nalfurafine treatment and unit drug dose on fentanyl/cocaine mixture choice (%)  Mixed-effects model (REML)  Figure 3A: Effect of nalfurafine treatment on fentanyl/cocaine mixture choice (%)  Mixed-effects model (REML) | **Unit Fent/Coc Dose**  **Nalfurafine Dose**  Interaction  Nalfurafine Dose | **F_1.61,16.09_=85.71**  **F_2.32,23.17_=5.61**  F_3.71,33.72_=2.02  F_1.73,16.75_=1.32 | **<0.0001**  **0.0080**  0.1178  0.29 |
| Figure 3B: Effect of nalfurafine treatment and unit drug dose on fentanyl choice (%)  Mixed-effects model (REML) | **Unit Fentanyl Dose**  Nalfurafine Dose  Interaction | **F_1.75,19.21_=105.6**  F_1.77,19.45_=0.80  F_2.83,17.00_=1.91 | **<0.0001**  0.4501  0.1686 |
| Figure 3C: Effect of nalfurafine treatment and unit drug dose on cocaine choice (%)  Mixed-effects model (REML)  Figure 3C: Effect of nalfurafine treatment on cocaine choice (%)  Mixed-effects model (REML) | **Unit Cocaine Dose**  **Nalfurafine Dose**  Interaction  **Nalfurafine Dose** | **F_1.56,12.44_=151.4**  **F_2.88,23.05_=5.91**  F_4.26,28.47_=2.46  **F_2.84,19.86_=5.83** | **<0.0001**  **0.0041**  0.0652  **0.0056** |
| Figure 3D: Effect of nalfurafine treatment and unit drug dose on total choices/component (when drug=fentanyl/cocaine mixture)  Mixed-effects model (REML) | **Unit Fent/Coc Dose**  **Nalfurafine Dose**  **Interaction** | **F_2.38,23.76_=56.99**  **F_1.45,14.47_=5.95**  **F_4.17,39.97_=3.96** | **<0.0001**  **0.0194**  **0.0077** |
| Figure 3E: Effect of nalfurafine treatment and unit drug dose on total choices/component (when drug=fentanyl)  Mixed-effects model (REML) | **Unit Fentanyl Dose**  Nalfurafine Dose  Interaction | **F_1.10,12.14_=230.6**  F_1.24,13.67_=0.37  F_2.22,13.33_=1.52 | **<0.0001**  0.5985  0.2554 |
| Figure 3F: Effect of nalfurafine treatment and unit drug dose on total choices/component (when drug=cocaine)  Mixed-effects model (REML) | **Unit Cocaine Dose**  Nalfurafine Dose  Interaction | **F_1.93,15.42_=32.05**  F_1.53,12.20_=0.77  F_2.72,18.35_=2.14 | **<0.0001**  0.4511  0.1348 |
| Figure 3G: Effect of nalfurafine and reinforcer type on choices/session (when drug=fentanyl/cocaine mixture)  Mixed-effects model (REML) | **Reinforcer Type**  **Nalfurafine Dose**  **Interaction** | **F_1.58,15.84_=41.34**  **F_1.45,14.47_=5.95**  **F_2.70,25.69_=5.35** | **<0.0001**  **0.0194**  **0.0066** |
| Figure 3H: Effect of nalfurafine and reinforcer type on choices/session (when drug=fentanyl)  Mixed-effects model (REML) | **Reinforcer Type**  Nalfurafine Dose  Interaction | **F_1.01,11.06_=112.4**  F_1.24,13.67_=0.44  F_1.90,9.44_=2.87 | **<0.0001**  0.5628  0.1083 |
| Figure 3I: Effect of nalfurafine and reinforcer type on choices/session (when drug=cocaine)  Mixed-effects model (REML) | **Reinforcer Type**  Nalfurafine Dose  **Interaction** | **F_1.19,9.55_=78.41**  F_1.53,12.20_=0.28  **F_2.78,18.05_=8.29** | **<0.0001**  0.7003  **0.0013** |
|  |  |  |  |
| Figure 4A: Effect of VZTK35 treatment and unit drug dose on fentanyl/cocaine mixture choice (%)  Mixed-effects model (REML) | **Unit Fent/Coc Dose**  **VZTK35 Dose**  **Interaction** | **F_3.21,25.66_=38.20**  **F_1.97,15.79_=37.57**  **F_2.98,18.61_=4.72** | **<0.0001**  **<0.0001**  **0.0130** |
| Figure 4B: Effect of VZTK35 treatment and unit drug dose on fentanyl choice (%)  Mixed-effects model (REML) | **Unit Fentanyl Dose**  VZTK35 Dose  Interaction | **F_2.44,19.52_=35.21**  F_1.17,9.36_=1.07  F_2.94,9.56_=1.25 | **<0.0001**  0.3411  0.3441 |
| Figure 4C: Effect of VZTK35 treatment and unit drug dose on cocaine choice (%)  Mixed-effects model (REML)  Figure 4C: Effect of VZTK35 treatment on cocaine choice (%)  RM one-way ANOVA | **Unit Cocaine Dose**  **VZTK35 Dose**  Interaction  **VZTK35 Dose** | **F_2.13,12.79_=34.06**  **F_1.78,10.65_=5.32**  F_2.56,13.77_=1.82  **F_1.73,10.38_=6.90** | **<0.0001**  **0.0278**  0.1943  **0.0145** |
| Figure 4D: Effect of VZTK35 treatment and unit drug dose on total choices/component (when drug=fentanyl/cocaine mixture)  Mixed-effects model (REML) | **Unit Fent/Coc Dose**  **VZTK35 Dose**  **Interaction** | **F_1.81,14.45_=13.91**  **F_1.38,11.04_=26.83**  **F_4.09,30.13_=7.15** | **0.0006**  **0.0001**  **0.0003** |
| Figure 4E: Effect of VZTK35 treatment and unit drug dose on total choices/component (when drug=fentanyl)  RM two-way ANOVA | **Unit Fentanyl Dose**  **VZTK35 Dose**  **Interaction** | **F_2.11,16.89_=41.02**  **F_1.89,15.08_=22.25**  **F_3.46,27.66_=15.53** | **<0.0001**  **<0.0001**  **<0.0001** |
| Figure 4F: Effect of VZTK35 treatment and unit drug dose on total choices/component (when drug=cocaine)  RM two-way ANOVA | **Unit Cocaine Dose**  **VZTK35 Dose**  **Interaction** | **F_1.44,8.63_=12.85**  **F_1.51,9.05_=32.36**  **F_3.32,19.94_=8.51** | **0.0040**  **0.0001**  **0.0006** |
| Figure 4G: Effect of VZTK35 and reinforcer type on choices/session (when drug=fentanyl/cocaine mixture)  RM two-way ANOVA | **Reinforcer Type**  **VZTK35 Dose**  **Interaction** | **F_1.40,11.23_=19.05**  **F_1.86,14.92_=33.21**  **F_2.06,16.48_=19.37** | **0.0005**  **<0.0001**  **<0.0001** |
| Figure 4H: Effect of VZTK35 and reinforcer type on choices/session (when drug=fentanyl)  RM two-way ANOVA | **Reinforcer Type**  **VZTK35 Dose**  **Interaction** | **F_1.71,13.65_=70.80**  **F_1.89,15.08_=25.25**  **F_2.33,18.60_=13.84** | **<0.0001**  **<0.0001**  **0.0001** |
| Figure 4I: Effect of VZTK35 and reinforcer type on choices/session (when drug=cocaine)  RM two-way ANOVA | **Reinforcer Type**  **VZTK35 Dose**  **Interaction** | **F_1.32,7.91_=27.30**  **F_1.51,9.05_=32.36**  **F_1.52,9.09_=9.85** | **0.0005**  **0.0001**  **0.0074** |
|  |  |  |  |
| Figure 5A: Effect of nalfurafine with nor-BNI pretreatment and unit drug dose on fentanyl/cocaine choice (%)  Mixed-effects model (REML)  Figure 5A: Effect of nalfurafine with nor-BNI pretreatment on fentanyl/cocaine choice (%)  Mixed-effects model (REML) | **Unit Fent/Coc Dose**  **nor-BNI**  Interaction  nor-BNI | **F_2.15,21.51_=63.49**  **F_1.28,12.78_=5.01**  F_2.00,8.49_=2.31  F_1.22,7.34_=3.01 | **<0.0001**  **0.0366**  0.1583  0.1214 |
| Figure 5B: Effect of nalfurafine with nor-BNI pretreatment and unit drug dose on total choices/component  Mixed-effects model (REML) | **Unit Fent/Coc Dose**  nor-BNI  **Interaction^#^** | **F_1.93,19.25_=41.42**  F_1.01,10.08_=2.74  **F_2.44,12.22_=4.99** | **<0.0001**  0.1287  **0.0215** |
| Figure 5C: Effect of nalfurafine with nor-BNI pretreatment and reinforcer type on choices/session  Mixed-effects model (REML) | **Reinforcer Type**  nor-BNI  **Interaction^#^** | **F_1.22,12.21_=64.99**  F_1.01,10.08_=2.74  **F_2.03,8.12_=4.81** | **<0.0001**  0.1287  **0.0416** |
| Figure 5D: Effect of VZTK35 with nor-BNI pretreatment and unit drug dose on fentanyl/cocaine choice (%)  Mixed-effects model (REML) | **Unit Fent/Coc Dose**  **nor-BNI**  **Interaction** | **F_2.73,19.10_=59.16**  **F_1.67,11.65_=53.01**  **F_2.62,13.11_=7.47** | **<0.0001**  **<0.0001**  **0.0045** |
| Figure 5E: Effect of VZTK35 with nor-BNI pretreatment and unit drug dose on total choices/component  Mixed-effects model (REML) | **Unit Fent/Coc Dose**  **nor-BNI**  **Interaction** | **F_2.22,15.51_=26.51**  **F_1.23,8.63_=57.57**  **F_2.53,14.54_=15.40** | **<0.0001**  **<0.0001**  **0.0001** |
| Figure 5F: Effect of nalfurafine with nor-BNI pretreatment and reinforcer type on choices/session  Mixed-effects model (REML) | **Reinforcer Type**  **nor-BNI**  **Interaction** | **F_1.09,7.60_=40.06**  **F_1.23,8.63_=58.00**  **F_1.97,10.84_=21.80** | **0.0002**  **<0.0001**  **0.0002** |
|  |  |  |  |
| Figure 6A: Effect of naltrexone and unit drug dose on fentanyl/cocaine choice (%)  Mixed-effects model (REML) | **Unit Fent/Coc Dose**  Naltrexone Dose  Interaction | **F_1.43,12.82_=53.02**  F_1.45,13.03_=3.10  F_2.03,16.71_=0.82 | **<0.0001**  0.0908  0.4605 |
| Figure 6B: Effect of naltrexone and unit drug dose on total choices/component  Mixed-effects model (REML) | **Unit Fent/Coc Dose**  Naltrexone Dose  Interaction | **F_2.24,20.14_=18.68**  F_1.12,10.07_=1.43  F_2.73,22.89_=1.51 | **<0.0001**  0.2646  0.2408 |
| Figure 6C: Effect of naltrexone and reinforcer type on choices/session  Mixed-effects model (REML) | **Reinforcer Type**  Naltrexone Dose  Interaction | **F_1.14,10.27_=60.57**  F_1.12,10.07_=1.43  F_1.83,15.10_=2.01 | **<0.0001**  0.2646  0.1708 |

**^#^Dunnett-corrected post hoc comparisons revealed no significant pairwise differences.**

## Supplemental Table S3. Statistical details of sex comparisons in main text figures (GraphPad Prism Version 10.3 for Windows)

| **Figure number** | **Factor name** | **Values** | ***p*-value** |
| --- | --- | --- | --- |
| *Figure 1A: Effect of varying drug available within-session and sex on drug choice (%)  Mixed-effects model (REML) | **Drug Available**  Sex  Interaction | **F_1.53,6.90_=9.88**  F_1,5_=0.63  F_2,9_=2.03 | **0.0118**  0.4642  0.1877 |
| *Figure 1B: Effect of varying drug available within-session and sex on total choices/component  Mixed-effects model (REML) | Drug Available  Sex  Interaction | F_1.08,4.85_=2.19  F_1,5_=0.008  F_2,9_=0.06 | 0.2023  0.9304  0.9393 |
|  |  |  |  |
| *Figure 2A: Effect of varying drug or food FR requirements and sex on fentanyl/cocaine choice (%)  RM two-way ANOVA | **Cost (FR)**  Sex  Interaction | **F_1.87,9.36_=26.47**  F_1,5_=5.43  F_3,15_=0.05 | **0.0002**  0.0673  0.9841 |
| *Figure 2B: Effect of varying drug or food FR requirements and sex on total choices/component  RM two-way ANOVA | Cost (FR)  Sex  Interaction | F_1.55,7.74_=1.20  F_1,5_=0.002  F_3,15_=0.09 | 0.3360  0.9639  0.9657 |
| Figure 2C: Effect of varying drug or food FR requirements and sex on choices/session by reinforcer type  Total Choices  RM two-way ANOVA  Food Choices  RM two-way ANOVA  Drug Choices  RM two-way ANOVA | Cost (FR)  Sex  Interaction  **Cost (FR)**  Sex  Interaction  **Cost (FR)** Sex  Interaction | F_1.55,7.74_=1.20  F_1,5_=0.002  F_3,15_=0.09  **F_1.75,8.73_=10.53**  F_1,5_=0.52  F_3,15_=0.13  **F_1.80,9.0_=21.90**  F_1,5_=0.99  F_3,15_=0.12 | 0.3360  0.9639  0.9657  **0.0055**  0.5051  0.9386  **0.0004**  0.3659  0.9441 |
|  |  |  |  |
| *Figure 3A: Effect of nalfurafine treatment and sex on fentanyl/cocaine mixture choice (%)  Mixed-effects model (REML) | Nalfurafine Dose  **Sex**  Interaction | F_1.60,18.67_=1.34  **F_1,35_=18.62**  F_3,35_=0.82 | 0.2806  **0.0001**  0.4927 |
| *Figure 3B: Effect of nalfurafine treatment and sex on fentanyl choice (%)  Mixed-effects model (REML) | Nalfurafine Dose  Sex  Interaction | F_1.87,11.21_=1.03  F_1,10_=0.72  F_4,24_=1.68 | 0.3825  0.4170  0.1869 |
| *Figure 3C: Effect of nalfurafine treatment and sex on cocaine choice (%)  Mixed-effects model (REML) | **Nalfurafine Dose**  **Sex**  Interaction | **F_2.79,21.65_=5.60**  **F_1,31_=10.50**  F_4,31_=0.43 | **0.0061**  **0.0029**  0.7860 |
| *Figure 3D: Effect of nalfurafine treatment and sex on total choices/component (when drug=fentanyl/cocaine mixture)  Mixed-effects model (REML) | **Nalfurafine Dose**  **Sex**  **Interaction** | **F_1.71,14.84_=8.52**  **F_1,9_=7.32**  **F_3,26_=7.84** | **0.0046**  **0.0242**  **0.0007** |
| *Figure 3E: Effect of nalfurafine treatment and sex on total choices/component (when drug=fentanyl)  Mixed-effects model (REML) | **Nalfurafine Dose**  Sex  **Interaction^#^** | **F_1.32,7.90_=6.86**  F_1,10_=3.42  **F_4,24_=4.65** | **0.0257**  0.0942  **0.0064** |
| *Figure 3F: Effect of nalfurafine treatment and sex on total choices/component (when drug=cocaine)  Mixed-effects model (REML) | Nalfurafine Dose  Sex  Interaction | F_1.37,8.23_=0.78  F_1,7_=0.30  F_4,24_=1.33 | 0.4436  0.6024  0.2892 |
| Figure 3G: Effect of nalfurafine and sex on total choices/session by reinforcer type (when drug=fentanyl/cocaine mixture)  Total Choices  Mixed-effects model (REML)  Food Choices  Mixed-effects model (REML)  Drug Choices  Mixed-effects model (REML) | **Nalfurafine Dose**  **Sex**  **Interaction**  **Nalfurafine Dose**  **Sex**  **Interaction**  Nalfurafine Dose  Sex  Interaction | **F_1.71,14.84_=8.52**  **F_1,9_=7.32**  **F_3,26_=7.84**  **F_2.54,22.00_=17.69**  **F_1,9_=30.16**  **F_3,26_=8.51**  F_2.65,22.94_=0.63  F_1,9_=1.59  F_3,26_=1.22 | **0.0046**  **0.0242**  **0.0007**  **<0.0001**  **0.0004**  **0.0004**  0.5817  0.2393  0.3226 |
| Figure 3H: Effect of nalfurafine and sex on choices/session by reinforcer type (when drug=fentanyl)  Total Choices  Mixed-effects model (REML)  Food Choices  Mixed-effects model (REML)  Drug Choices  Mixed-effects model (REML) | Nalfurafine Dose  Sex  Interaction  Nalfurafine Dose  Sex  Interaction  Nalfurafine Dose  Sex  Interaction | F_1.25,7.49_=0.74  F_1,10_=2.19  F_4,24_=0.90  F_1.59,9.57_=2.13  F_1,10_=0.71  F_4,24_=1.63  F_2.38,14.29_=3.09  F_1,10_=0.24  F_4,24_=2.00 | 0.4479  0.1694  0.4808  0.1749  0.4205  0.1995  0.0701  0.6373  0.1270 |
| Figure 3I: Effect of nalfurafine and sex on choices/session by reinforcer type (when drug=cocaine)  Total Choices  Mixed-effects model (REML)  Food Choices  Mixed-effects model (REML)  Drug Choices  Mixed-effects model (REML) | Nalfurafine Dose  Sex  Interaction  **Nalfurafine Dose**  **Sex**  Interaction  **Nalfurafine Dose**  **Sex**  Interaction | F_1.37,8.23_=0.78  F_1,7_=0.30  F_4,24_=1.33  **F_2.78,16.67_=5.71**  **F_1,7_=6.49**  F_4,24_=0.45  **F_2.42,18.75_=5.76**  **F_1,31_=9.13**  F_4,31_=0.35 | 0.4436  0.6024  0.2892  **0.0079**  **0.0383**  0.7723  **0.0083**  **0.0050**  0.8408 |
|  |  |  |  |
| *Figure 4A: Effect of VZTK35 treatment and sex on fentanyl/cocaine mixture choice (%)  Mixed-effects model (REML) | **VZTK35 Dose**  Sex  Interaction | **F_1.56,10.11_=34.09**  F_1,7_=2.47  F_2,13_=1.65 | **<0.0001**  0.1603  0.2302 |
| *Figure 4B: Effect of VZTK35 treatment and sex on fentanyl choice (%)  Mixed-effects model (REML) | **VZTK35 Dose**  Sex  Interaction | **F_1.31,7.22_=8.87**  F_1,7_=1.01  F_2,11_=2.51 | **0.0161**  0.3477  0.1262 |
| *Figure 4C: Effect of VZTK35 treatment and sex on cocaine choice (%)  RM two-way ANOVA | **VZTK35 Dose**  Sex  Interaction | **F_1.85,9.25_=6.24**  F_1,5_=0.03  F_2,10_=0.39 | **0.0206**  0.8651  0.6875 |
| *Figure 4D: Effect of VZTK35 treatment and sex on total choices/component (when drug=fentanyl/cocaine mixture)  Mixed-effects model (REML) | **VZTK35 Dose**  **Sex**  **Interaction^#^** | **F_1.36,8.85_=33.75**  **F_1,7_=5.65**  **F_2,13_=3.91** | **0.0002**  **0.0492**  **0.0469** |
| *Figure 4E: Effect of VZTK35 treatment and sex on total choices/component (when drug=fentanyl)  RM two-way ANOVA | **VZTK35 Dose**  Sex  Interaction | **F_1.83,12.83_=23.16**  F_1,7_=1.74  F_2,14_=0.31 | **<0.0001**  0.2282  0.7393 |
| *Figure 4F: Effect of VZTK35 treatment and sex on total choices/component (when drug=cocaine)  RM two-way ANOVA | **VZTK35 Dose**  Sex  Interaction | **F_1.43,7.12_=30.59**  F_1,5_=2.35  F_2,10_=0.67 | **0.0005**  0.1860  0.5321 |
| Figure 4G: Effect of VZTK35 and sex on choices/session by reinforcer type (when drug=fentanyl/cocaine mixture)  Total Choices  RM two-way ANOVA  Food Choices  RM two-way ANOVA  Drug Choices  RM two-way ANOVA | **VZTK35 Dose**  **Sex**  **Interaction^#^**  **VZTK35 Dose**  **Sex**  **Interaction**  VZTK35 Dose  Sex  Interaction | **F_1.74,12.21_=45.37**  **F_1,7_=5.61**  **F_2,14_=4.12**  **F_1.93,13.53_=52.86**  **F_1,7_=11.95**  **F_2,14_=7.53**  F_1.64,11.51_=0.39  F_1,7_=0.03  F_2,14_=3.01 | **<0.0001**  **0.0497**  **0.0392**  **<0.0001**  **0.0106**  **0.0060**  0.6465  0.8643  0.0820 |
| Figure 4H: Effect of VZTK35 and sex on choices/session by reinforcer type (when drug=fentanyl)  Total Choices  RM two-way ANOVA  Food Choices  RM two-way ANOVA  Drug Choices  RM two-way ANOVA | **VZTK35 Dose**  Sex  Interaction  **VZTK35 Dose**  Sex  Interaction  **VZTK35 Dose**  Sex  **Interaction^#^** | **F_1.83,12.83_=23.16**  F_1,7_=1.74  F_2,14_=0.31  **F_1.99,13.93_=18.12**  F_1,7_=0.56  F_2,14_=0.34  **F_1.22,8.55_=10.92**  F_1,7_=1.86  **F_2,14_=4.93** | **<0.0001**  0.2282  0.7393  **0.0001**  0.4786  0.7147  **0.0079**  0.2148  **0.0239** |
| Figure 4I: Effect of VZTK35 and sex on choices/session by reinforcer type (when drug=cocaine)  Total Choices  RM two-way ANOVA  Food Choices  RM two-way ANOVA  Drug Choices  RM two-way ANOVA | **VZTK35 Dose**  Sex  Interaction  **VZTK35 Dose**  Sex  Interaction  VZTK35 Dose  Sex  Interaction | **F_1.43,7.12_=30.59**  F_1,5_=2.35  F_2,10_=0.67  **F_1.21,6.07_=17.20**  F_1,5_=0.1974  F_2,10_=0.02  F_1.45,7.23_=1.65  F_1,5_=1.99  F_2,10_=0.52 | **0.0005**  0.1860  0.5321  **0.0049**  0.6754  0.9790  0.2504  0.2170  0.6088 |
|  |  |  |  |
| *Figure 5A: Effect of nalfurafine with nor-BNI pretreatment and sex on fentanyl/cocaine choice (%)  Mixed-effects model (REML) | **nor-BNI**  Sex  **Interaction^#^** | **F_1.20,5.98_=8.53**  F_1,9_=0.02  **F_2,10_=4.44** | **0.0241**  0.8961  **0.0418** |
| *Figure 5B: Effect of nalfurafine with nor-BNI pretreatment and sex on total choices/component  Mixed-effects model (REML) | **nor-BNI**  Sex  **Interaction** | **F_1.02,5.10_=7.28**  F_1,9_=3.28  **F_2,10_=8.77** | **0.0417**  0.1034  **0.0063** |
| Figure 5C: Effect of nalfurafine with nor-BNI pretreatment and sex on choices/session by reinforcer type  Total Choice  Mixed-effects model (REML)  Food Choice  Mixed-effects model (REML)  Drug Choice  Mixed-effects model (REML) | **nor-BNI**  Sex  **Interaction**  **nor-BNI**  **Sex**  **Interaction**  nor-BNI  Sex  Interaction | **F_1.02,5.10_=7.28**  F_1,9_=3.28  **F_2,10_=8.77**  **F_1.39,6.94_=34.14**  **F_1,9_=10.06**  **F_2,10_=26.63**  F_1.18,11.24_=0.34  F_1,19_=0.12  F_2,19_=0.30 | **0.417**  0.1034  **0.0063**  **0.0004**  **0.0113**  **<0.0001**  0.6079  0.7338  0.7468 |
| *Figure 5D: Effect of VZTK35 with nor-BNI pretreatment and sex on fentanyl/cocaine choice (%)  Mixed-effects model (REML) | **nor-BNI**  Sex  Interaction | **F_1.14,5.68_=33.68**  F_1,6_=3.19  F_2,10_=1.54 | **0.0012**  0.1244  0.2620 |
| *Figure 5E: Effect of VZTK35 with nor-BNI pretreatment and sex on total choices/component  Mixed-effects model (REML) | **nor-BNI**  Sex  Interaction | **F_1.06,5.29_=49.19**  F_1,6_=0.64  F_2,10_=0.60 | **0.0007**  0.4545  0.5670 |
| Figure 5F: Effect of nalfurafine with nor-BNI pretreatment and sex on choices/session by reinforcer type  Total Choices  Mixed-effects model (REML)  Food Choices  Mixed-effects model (REML)  Drug Choices  Mixed-effects model (REML) | **nor-BNI**  Sex  Interaction  **nor-BNI**  Sex  **Interaction^#^**  nor-BNI  Sex  Interaction | **F_1.06,5.29_=49.19**  F_1,6_=0.64  F_2,10_=0.60  **F_1.96,9.81_=108.1**  F_1,6_=4.20  **F_2,10_=6.31**  F_1.40,7.00_=1.69  F_1,6_=0.25  F_2,10_=1.14 | **0.0007**  0.4545  0.5670  **<0.0001**  0.0862  **0.0169**  0.2448  0.6363  0.3574 |
|  |  |  |  |
| *Figure 6A: Effect of naltrexone and sex on fentanyl/cocaine choice (%)  Mixed-effects model (REML) | Naltrexone Dose  Sex  Interaction | F_1.27,9.55_=2.91  F_1,8_=1.88  F_2,15_=1.58 | 0.1158  0.2080  0.2386 |
| *Figure 6B: Effect of naltrexone and sex on total choices/component  Mixed-effects model (REML) | Naltrexone Dose  **Sex**  Interaction | F_1.15,8.63_=1.69  **F_1,8_=6.23**  F_2,15_=2.61 | 0.2318  **0.0372**  0.1069 |
| Figure 6C: Effect of naltrexone and sex on choices/session by reinforcer type  Total Choices  Mixed-effects model (REML)  Food Choices  Mixed-effects model (REML)  Drug Choices  Mixed-effects model (REML) | Naltrexone Dose  **Sex**  Interaction  Naltrexone Dose  Sex  Interaction  Naltrexone Dose  Sex  Interaction | F_1.15,8.63_=1.69  **F_1,8_=6.23**  F_2,15_=2.61  F_1.20,8.98_=2.31  F_1,8_=4.91  F_2,15_=1.48  F_1.52,11.40_=1.29  F_1,8_=0.1278  F_2,15_=1.93 | 0.2318  **0.0372**  0.1069  0.1621  0.0575  0.2588  0.3008  0.7300  0.1791 |

***Sex comparisons of % drug choice and total choices/component were collapsed by unit drug dose to facilitate analysis.**

**^#^Šídák-corrected post hoc comparisons revealed no significant pairwise differences.**

## Supplemental Table S4. Statistical details of supplementary tables and figures (GraphPad Prism Version 10.3 for Windows)

| **Figure number** | **Factor name** | **Values** | ***p*-value** |
| --- | --- | --- | --- |
| Figure S1A: Baseline % 1:94 fentanyl/cocaine mixture choice  RM one-way ANOVA | **Unit Fent/Coc Dose** | **F_2.06,20.61_=64.89** | **<0.0001** |
| Figure S1B: Baseline # choices per session  RM one-way ANOVA | **Unit Fent/Coc Dose** | **F_2.29,22.90_=36.82** | **<0.0001** |
|  |  |  |  |
| Figure S2A: Effect of varying drug available between cohorts and unit drug dose on drug choice (%)  Mixed-effects model (REML) | **Unit Drug Dose**  **Drug Available** | **F_5,123_=126.3**  **F_2,29_=12.99** | **<0.0001**  **<0.0001** |
| Figure S2B: Effect of varying drug available between cohorts and unit drug dose on total choices/component  Mixed-effects model (REML) | **Unit Drug Dose**  **Drug Available** | **F_5,123_=85.94**  **F_2,29_=18.42** | **<0.0001**  **<0.0001** |
|  |  |  |  |
| Figure S3A: Effect of nalfurafine treatment and unit drug dose on day 1 fentanyl/cocaine mixture choice (%)  Mixed-effects model (REML) | **Unit Fent/Coc Dose**  Nalfurafine Dose  Interaction | **F_2.02,20.15_=59.64**  F_2.40,23.95_=0.50  F_4.01,32.04_=0.88 | **<0.0001**  0.6449  0.4845 |
| Figure S3B: Effect of nalfurafine treatment and unit drug dose on averaged days 4&5 fentanyl/cocaine mixture choice (%)  Please see Table S1 Figure 3A |  |  |  |
| Figure S3C: Effect of nalfurafine treatment and unit drug dose on day 1 total choices/component (when drug=fentanyl/cocaine mixture)  Mixed-effects model (REML) | **Unit Fent/Coc Dose**  **Nalfurafine Dose**  **Interaction** | **F_2.38,23.77_=51.54**  **F_1.35,13.52_=12.99**  **F_2.76,26.49_=6.84** | **<0.0001**  **0.0017**  **0.0018** |
| Figure S3D: Effect of nalfurafine treatment and unit drug dose on averaged days 4&5 total choices/component (when drug=fentanyl/cocaine mixture)  Please see Table S1 Figure 3D |  |  |  |
|  |  |  |  |
| Figure S4A: Effect of nalfurafine treatment and unit drug dose on day 1 fentanyl choice (%)  RM two-way ANOVA | **Unit Fentanyl Dose**  Nalfurafine Dose  Interaction | **F_1.57,10.96_=54.13**  F_2.56,17.90_=2.21  F_4.07,28.47_=1.47 | **<0.0001**  0.1292  0.2367 |
| Figure S4B: Effect of nalfurafine treatment and unit drug dose on averaged days 4&5 fentanyl choice (%)  Please see Table S1 Figure 3B |  |  |  |
| Figure S4C: Effect of nalfurafine treatment and unit drug dose on day 1 total choices/component (when drug=fentanyl)  RM two-way ANOVA | **Unit Fentanyl Dose**  Nalfurafine Dose  Interaction | **F_1.03,7.20_=186**  F_1.99,13.94_=2.11  F_2.28,15.99_=2.10 | **<0.0001**  0.1582  0.1503 |
| Figure S4D: Effect of nalfurafine treatment and unit drug dose on averaged days 4&5 total choices/component (when drug=fentanyl)  Please see Table S1 Figure 3E |  |  |  |
|  |  |  |  |
| Figure S5A: Effect of nalfurafine treatment and unit drug dose on day 1 cocaine choice (%)  Mixed-effects model (REML) | **Unit Cocaine Dose**  Nalfurafine Dose  Interaction | **F_2.04,14.25_=74.59**  F_2.27,15.88_=1.14  F_3.84,25.69_=1.16 | **<0.0001**  0.3519  0.3501 |
| Figure S5B: Effect of nalfurafine treatment and unit drug dose on averaged days 4&5 cocaine choice (%)  Please see Table S1 Figure 3C |  |  |  |
| Figure S5C: Effect of nalfurafine treatment and unit drug dose on day 1 total choices/component (when drug=cocaine)  RM two-way ANOVA | **Unit Cocaine Dose**  Nalfurafine Dose  Interaction | **F_1.95,13.63_=33.21**  F_1.96,13.70_=2.42  F_2.83,19.84_=1.38 | **<0.0001**  0.1268  0.2780 |
| Figure S5D: Effect of nalfurafine treatment and unit drug dose on averaged days 4&5 total choices/component (when drug=cocaine)  Please see Table S1 Figure 3F |  |  |  |
|  |  |  |  |
| Figure S6A: Effect of VZTK35 treatment and unit drug dose on day 1 fentanyl/cocaine mixture choice (%)  Mixed-effects model (REML) | **Unit Fent/Coc Dose**  VZTK35 Dose  Interaction | **F_4.00,32.00_=15.30**  F_8.00,23.00_=1.68 | **<0.001**  ND  0.1580 |
| Figure S6B: Effect of VZTK35 treatment and unit drug dose on averaged days 4&5 fentanyl/cocaine mixture choice (%)  Please see Table S1 Figure 4A |  |  |  |
| Figure S6C: Effect of VZTK35 treatment and unit drug dose on day 1 total choices/component (when drug=fentanyl/cocaine mixture)  Mixed-effects model (REML) | **Unit Fent/Coc Dose**  **VZTK35 Dose**  **Interaction** | **F_2.18,17.41_=15.45**  **F_1.39,11.15_=64.63**  **F_3.14,23.18_=6.60** | **0.0001**  **<0.0001**  **0.0020** |
| Figure S6D: Effect of VZTK35 treatment and unit drug dose on averaged days 4&5 total choices/component (when drug=fentanyl/cocaine mixture)  Please see Table S1 Figure 4D |  |  |  |
|  |  |  |  |
| Figure S7A: Effect of VZTK35 treatment and unit drug dose on day 1 fentanyl choice (%)  Mixed-effects model (REML) | **Unit Fentanyl Dose**  VZTK35 Dose  **Interaction** | **F_4.00,32.00_=25.94**  **F_8.00,18.00_=5.86** | **<0.0001**  ND  **0.0009** |
| Figure S7B: Effect of VZTK35 treatment and unit drug dose on averaged days 4&5 fentanyl choice (%)  Please see Table S1 Figure 4B |  |  |  |
| Figure S7C: Effect of VZTK35 treatment and unit drug dose on day 1 total choices/component (when drug=fentanyl)  RM two-way ANOVA | **Unit Fentanyl Dose**  **VZTK35 Dose**  **Interaction** | **F_1.75,14.01_=32.80**  **F_1.41,11.25_=39.20**  **F_2.51,20.09_=11.78** | **<0.0001**  **<0.0001**  **0.0002** |
| Figure S7D: Effect of VZTK35 treatment and unit drug dose on averaged days 4&5 total choices/component (when drug=fentanyl)  Please see Table S1 Figure 4E |  |  |  |
|  |  |  |  |
| Figure S8A: Effect of VZTK35 treatment and unit drug dose on day 1 cocaine choice (%)  Mixed-effects model (REML) | **Unit Cocaine Dose**  **VZTK35 Dose**  Interaction | **F_1.60,9.59_=37.46**  **F_1.39,8.36_=5.91**  F_1.95,4.40_=4.17 | **<0.0001**  **0.0325**  0.0969 |
| Figure S8B: Effect of VZTK35 treatment and unit drug dose on averaged days 4&5 cocaine choice (%)  Please see Table S1 Figure 4C |  |  |  |
| Figure S8C: Effect of VZTK35 treatment and unit drug dose on day 1 total choices/component (when drug=cocaine)  RM two-way ANOVA | **Unit Cocaine Dose**  **VZTK35 Dose**  **Interaction** | **F_2.72,16.34_=3.35**  **F_1.19,7.11_=38.56**  **F_2.93,17.55_=6.34** | **0.0482**  **0.0003**  **0.0044** |
| Figure S8D: Effect of VZTK35 treatment and unit drug dose on averaged days 4&5 total choices/component (when drug=cocaine)  Please see Table S1 Figure 4F |  |  |  |
|  |  |  |  |
| Figure S9A: Effect of nalfurafine with nor-BNI pretreatment and unit drug dose on day 1 fentanyl/cocaine choice (%)  Mixed-effects model (REML) | **Unit Fent/Coc Dose**  nor-BNI  Interaction | **F_1.89,17.01_=37.05**  F_1.37,12.31_=0.24  F_2.56,9.91_=0.79 | **<0.0001**  0.7053  0.5087 |
| Figure S9B: Effect of nalfurafine with nor-BNI pretreatment and unit drug dose on averaged days 4&5 fentanyl/cocaine choice (%)  Please See Table S1 Figure 5A |  |  |  |
| Figure S9C: Effect of nalfurafine with nor-BNI pretreatment and unit drug dose on day 1 total choices/component  Mixed-effects model (REML) | **Unit Fent/Coc Dose**  **nor-BNI**  **Interaction** | **F_2.92,29.23_=32.09**  **F_1.01,10.14_=8.65**  **F_2.50,12.51_=6.57** | **<0.0001**  **0.0144**  **0.0084** |
| Figure S9D: Effect of nalfurafine with nor-BNI pretreatment and unit drug dose on averaged days 4&5 total choices/component  Please see Table S1 Figure 5B |  |  |  |
|  |  |  |  |
| Figure S10A: Effect of VZTK35 with nor-BNI pretreatment and unit drug dose on day 1 fentanyl/cocaine choice (%)  Mixed-effects model (REML) | **Unit Fent/Coc Dose**  VZTK35 Dose  Interaction | **F_1.00,32.00_=12.80**  F_8.00,8.00_=2.69 | **<0.0001**  ND  0.0915 |
| Figure S10B: Effect of VZTK35 with nor-BNI pretreatment and unit drug dose on averaged days 4&5 fentanyl/cocaine choice (%)  Please see Table S1 Figure 5D |  |  |  |
| Figure S10C: Effect of VZTK35 with nor-BNI pretreatment and unit drug dose on day 1 total choices/component  Mixed-effects model (REML) | **Unit Fent/Coc Dose**  **VZTK35 Dose**  **Interaction** | **F_2.00,16.03_=29.42**  **F_1.94,15.53_=192.4**  **F_1.94,8.24_=9.55** | **<0.0001**  **<0.0001**  **0.0074** |
| Figure S10D: Effect of VZTK35 with nor-BNI pretreatment and unit drug dose on averaged days 4&5 total choices/component  Please see Table S1 Figure 5E |  |  |  |
|  |  |  |  |
| Figure S11A: Effect of naltrexone and unit drug dose on day 1 fentanyl/cocaine choice (%)  Mixed-effects model (REML) | **Unit Fent/Coc Dose**  Naltrexone Dose  Interaction | **F_1.60,14.37_=70.93**  F_1.76,15.80_=0.51  F_3.31,26.92_=1.06 | **<0.0001**  0.5882  0.3864 |
| Figure S11B: Effect of naltrexone and unit drug dose on averaged days 4&5 fentanyl/cocaine choice (%)  Please see Table S1 Figure 6A |  |  |  |
| Figure S11C: Effect of naltrexone and unit drug dose on day 1 total choices/component  Mixed-effects model (REML) | **Unit Fent/Coc Dose**  Naltrexone Dose  **Interaction** | **F_3.28,29.47_=14.40**  F_1.67,15.04_=1.83  **F_3.86,32.34_=4.15** | **<0.0001**  0.1968  **0.0086** |
| Figure S11D: Effect of naltrexone and unit drug dose on averaged days 4&5 total choices/component  Please see Table S1 Figure 6B |  |  |  |

ND: The p-value could not be determined; lack of choice resulted in undefined percentages (i.e., too many missing values)

## Supplemental Table S5. ED_50_ choice values, predicted additive Z_add_ values and empirically determined Z_mix_ values in µg/kg/infusion (95% confidence limits) during availability of fentanyl alone, cocaine alone, and a 1:94 fentanyl/cocaine mixture in male and female rats by training drug.

|  | Fentanyl ED_50_ | Cocaine ED_50_ | Z_mix_ | Z_add_ |
| --- | --- | --- | --- | --- |
| *Fentanyl-trained* | |  |  | 87.5 (80.8 – 94.2)^#^ |
|  | 1.9 (1.5 – 2.3)* | - | - |  |
| *Cocaine-trained* | - | 174.4 (134.7 – 225.8)* | - |  |
| *1:94 Fentanyl/Cocaine-trained* | |  |  |  |
|  | 0.6 (0.4 – 0.7) | 52.8 (40.3 – 69.1) | 53.3 (21.1 – 43.7) | - |

*ED_50_ value significantly different for fentanyl or cocaine alone when compared to ED_50_ value for that drug in 1:94 fentanyl/cocaine mixture baseline based on non-overlapping confidence limits.

^#^ Significant difference between Z_mix_ and Z_add_ based on non-overlapping confidence limits.

# Supplemental Figures

Figure S1. Baseline of 1:94 fentanyl/cocaine mixture-vs.-food choice in male (n=6) and female (n=5) rats. (**A**) Percent 1:94 fentanyl/cocaine mixture choice. Abscissa: Intravenous unit doses of fentanyl (bold) and cocaine (italics) in µg/kg/inf. (**B**) Total number of choices completed per component. Abscissa: Intravenous unit doses of fentanyl (bold) and cocaine (italics) in µg/kg/inf. (**C**) Total number of choices completed per session. Abscissa: Reinforcer type. All points represent the mean ± SEM. Filled symbols denote significant difference relative to the first component (0 µg/kg/inf fentanyl/cocaine), defined as p<0.05. See **Table S4** for statistics relevant to each panel.

Figure S2. Effects of varying the drug available between cohorts on choice in male and female rats (Fentanyl: male n=7, female n=5; Cocaine: n=5, female n=4; 1:94 fentanyl/cocaine: male n=6, female n=5). (**A**) Percent drug choice; drug is 1:94 fentanyl/cocaine mixture (circles), fentanyl alone (triangles), or cocaine alone (squares). Abscissa: Intravenous unit doses of fentanyl (bold) and cocaine (italics) in µg/kg/inf. (**B**) Total number of choices completed per component. Abscissa: Intravenous unit doses of fentanyl (bold) and cocaine (italics) in µg/kg/inf. (**C**) Isobologram for the interaction between fentanyl and cocaine at a 1:1 ED_50_ ratio based on the potency of each drug alone in a drug choice procedure. Ordinate: Cocaine Choice ED_50_ in µg/kg/inf. Abscissa: Fentanyl Choice ED_50_ in µg/kg/inf. All points represent the mean ± SEM. Filled symbols indicate significant difference between the 1:94 fentanyl/cocaine dose-effect function and that of a single substance (fentanyl or cocaine alone), defined as p<0.05. Asterisk denotes significant difference from predicted additivity. See **Table S4** for statistics relevant to each panel.

Figure S3. Effects of 5-day repeated subcutaneous injection of nalfurafine on 1:94 fentanyl/cocaine mixture-vs.-food choice in male and female rats [n=5-6 male, n=5 female]. (**A,B**) Percent 1:94 fentanyl/cocaine mixture choice on day 1 (**A**) and the average of days 4 & 5 (**B**). Abscissa: Intravenous unit doses of fentanyl (bold) and cocaine (italics) in µg/kg/inf. (**C,D**) Total number of choices completed per component on day 1 (**C**) and the average of days 4 & 5 (**D**). Abscissa: Intravenous unit doses of fentanyl (bold) and cocaine (italics) in µg/kg/inf. All points represent the mean ± SEM. Filled symbols denote significant difference relative to baseline (dashed lines), defined as p<0.05. See **Tables S1 and S4** for statistics relevant to each panel.

Figure S4. Effects of 5-day repeated subcutaneous injection of nalfurafine on fentanyl-vs.-food choice in male and female rats [n=4-7 male, n=4-5 female]. (**A,B**) Percent fentanyl choice on day 1 (**A**) and the average of days 4 & 5 (**B**). Abscissa: Intravenous unit doses of fentanyl in µg/kg/inf. (**C,D**) Total number of choices completed per component on day 1 (**C**) and the average of days 4 & 5 (**D**). Abscissa: Intravenous unit doses of fentanyl in µg/kg/inf. All points represent the mean ± SEM. Filled symbols denote significant difference relative to baseline (dashed lines), defined as p<0.05. See **Tables S1 and S4** for statistics relevant to each panel.

Figure S5. Effects of 5-day repeated subcutaneous injection of nalfurafine on cocaine-vs.-food choice in male and female rats [n=4-5 male, n=4 female]. (**A,B**) Percent cocaine choice on day 1 (**A**) and the average of days 4 & 5 (**B**). Abscissa: Intravenous unit doses of cocaine in µg/kg/inf. (**C,D**) Total number of choices completed per component on day 1 (**C**) and the average of days 4 & 5 (**D**). Abscissa: Intravenous unit doses of cocaine in µg/kg/inf. All points represent the mean ± SEM. Filled symbols denote significant difference relative to baseline (dashed lines), # denotes significant difference between nalfurafine (0.056 and 0.1 mg/kg) and baseline regardless of unit dose, defined as p<0.05. See **Tables S1 and S4** for statistics relevant to each panel.

Figure S6. Effects of 5-day repeated subcutaneous injection of VZTK35 on 1:94 fentanyl/cocaine mixture-vs.-food choice in male and female rats [n=3-4 male, n=5 female]. (**A,B**) Percent 1:94 fentanyl/cocaine mixture choice on day 1 (**A**) and the average of days 4 & 5 (**B**). Abscissa: Intravenous unit doses of fentanyl (bold) and cocaine (italics) in µg/kg/inf. (**C,D**) Total number of choices completed per component on day 1 (**C**) and the average of days 4 & 5 (**D**). Abscissa: Intravenous unit doses of fentanyl (bold) and cocaine (italics) in µg/kg/inf. All points represent the mean ± SEM. Filled symbols denote significant difference relative to baseline (dashed lines), defined as p<0.05. See **Tables S1 and S4** for statistics relevant to each panel.

Figure S7. Effects of 5-day repeated subcutaneous injection of VZTK35 on fentanyl-vs.-food choice in male and female rats [n=4 male, n=5 female]. (**A,B**) Percent fentanyl choice on day 1 (**A**) and the average of days 4 & 5 (**B**). Abscissa: Intravenous unit doses of fentanyl in µg/kg/inf. (**C,D**) Total number of choices completed per component on day 1 (**C**) and the average of days 4 & 5 (**D**). Abscissa: Intravenous unit doses of fentanyl in µg/kg/inf. All points represent the mean ± SEM. Filled symbols denote significant difference relative to baseline (dashed lines), defined as p<0.05. See **Tables S1 and S4** for statistics relevant to each panel.

Figure S8. Effects of 5-day repeated subcutaneous injection of VZTK35 on cocaine-vs.-food choice in male and female rats [n=3 male, n=4 female]. (**A,B**) Percent cocaine choice on day 1 (**A**) and the average of days 4 & 5 (**B**). Abscissa: Intravenous unit doses of cocaine in µg/kg/inf. (**C,D**) Total number of choices completed per component on day 1 (**C**) and the average of days 4 & 5 (**D**). Abscissa: Intravenous unit doses of cocaine in µg/kg/inf. All points represent the mean ± SEM. Filled symbols denote significant difference relative to baseline (dashed lines), # denotes significant difference between VZTK35 (0.032 mg/kg) and baseline regardless of unit dose, defined as p<0.05. See **Tables S1 and S4** for statistics relevant to each panel.

Figure S9. Effect of pretreatment with KOR antagonist nor-BNI prior to 5-day repeated subcutaneous injections of nalfurafine on 1:94 fentanyl/cocaine mixture-vs.-food choice in male (n=2-6) and female (n=5) rats. (**A,B**) Percent 1:94 fentanyl/cocaine mixture choice on day 1 (**A**) and the average of days 4 &5 (**B**). Abscissa: Intravenous unit doses of fentanyl (bold) and cocaine (italics) in µg/kg/inf. (**C,D**) Total number of choices completed per component on day 1 (**C**) and the average of days 4 & 5 (**D**). Abscissa: Intravenous unit doses of fentanyl (bold) and cocaine (italics) in µg/kg/inf. (**C&F**) Total number of choices completed per session. Abscissa: Reinforcer type. All points represent the mean ± SEM. Filled symbols denote significant difference relative to baseline (dashed lines), defined as p<0.05. See **Tables S1 and S4** for statistics relevant to each panel.

Figure S10. Effect of pretreatment with KOR antagonist nor-BNI prior to 5-day repeated subcutaneous injections of VZTK35 on 1:94 fentanyl/cocaine mixture-vs.-food choice in male (n=2-6) and female (n=5) rats. (**A,B**) Percent 1:94 fentanyl/cocaine mixture choice on day 1 (**A**) and the average of days 4 &5 (**B**). Abscissa: Intravenous unit doses of fentanyl (bold) and cocaine (italics) in µg/kg/inf. (**C,D**) Total number of choices completed per component on day 1 (**C**) and the average of days 4 & 5 (**D**). Abscissa: Intravenous unit doses of fentanyl (bold) and cocaine (italics) in µg/kg/inf. (**C&F**) Total number of choices completed per session. Abscissa: Reinforcer type. All points represent the mean ± SEM. Filled symbols denote significant difference relative to baseline (dashed lines), defined as p<0.05. See **Tables S1 and S4** for statistics relevant to each panel.

Figure S11. Effect of 5-day repeated subcutaneous injections of naltrexone on 1:94 fentanyl/cocaine mixture-vs.-food choice in male (n=4-5) and female (n=5) rats. (**A,B**) Percent 1:94 fentanyl/cocaine mixture choice on day 1 (**A**) and the average of days 4 & 5 (**B**). Abscissa: Intravenous unit doses of fentanyl (bold) and cocaine (italics) in µg/kg/inf. (**C,D**) Total number of choices completed per component on day 1 (**C**) and the average of days 4 & 5 (**D**). Abscissa: Intravenous unit doses of fentanyl (bold) and cocaine (italics) in µg/kg/inf. Filled symbols denote significant difference relative to baseline (dashed lines), defined as p<0.05. See **Tables S1 and S4** for statistics relevant to each panel.

**References**

St. Onge, C.M., Pagare, P.P., Zheng, Y., Arriaga, M., Stevens, D.L., Mendez, R.E., Poklis, J.L., Halquist, M.S., Selley, D.E., Dewey, W.L., Banks, M.L., Zhang, Y., 2024. Systematic Structure-Activity Relationship Study of Nalfurafine Analogues toward Development of Potentially Nonaddictive Pain Management Treatments. J Med Chem 67, 9552–9574. https://doi.org/10.1021/acs.jmedchem.4c00646
